# Visualization and quantification of *in vivo* homing kinetics of myeloid-derived suppressor cells in primary and metastatic cancer

**DOI:** 10.7150/thno.33275

**Published:** 2019-08-12

**Authors:** Sabrina H. L. Hoffmann, Dorothea I. Reck, Andreas Maurer, Birgit Fehrenbacher, Jaclyn E. Sceneay, Marilena Poxleitner, Hasan H. Öz, Walter Ehrlichmann, Gerald Reischl, Kerstin Fuchs, Martin Schaller, Dominik Hartl, Manfred Kneilling, Andreas Möller, Bernd J. Pichler, Christoph M. Griessinger

**Affiliations:** 1 Werner Siemens Imaging Center, Department of Preclinical Imaging and Radiopharmacy, Eberhard Karls University Tübingen, Tübingen, Germany; 2 iFIT-Cluster of Excellence, University of Tübingen, Germany; 3 Department of Dermatology, Eberhard Karls University Tübingen, Tübingen, Germany; 4 Tumor Microenvironment Laboratory, QIMR Berghofer Medical Research Institute, Herston, Australia; 5 Children's Hospital, University of Tübingen, Tübingen, Germany; 6 German Cancer Consortium (DKTK), Partner Site Tübingen; German Cancer Research Center (DKFZ), Heidelberg, Germany

**Keywords:** Myeloid-derived suppressor cells, cancer, cell tracking, positron emission tomography

## Abstract

Myeloid-derived suppressor cells (MDSCs) are immunosuppressive cells of the myeloid compartment and major players in the tumor microenvironment (TME). With increasing numbers of studies describing MDSC involvement in cancer immune escape, cancer metastasis and the dampening of immunotherapy responses, MDSCs are of high interest in current cancer therapy research. Although heavily investigated in the last decades, the *in vivo* migration dynamics of MDSC subpopulations in tumor- or metastases-bearing mice have not yet been studied extensively. Therefore, we have modified our previously reported intracellular cell labeling method and applied it to *in vitro* generated MDSCs for the quantitative *in vivo* monitoring of MDSC migration in primary and metastatic cancer. MDSC migration to primary cancers was further correlated to the frequency of endogenous MDSCs. **Methods:** Utilizing a ^64^Cu-labeled 1,4,7-triazacyclononane-triacetic acid (NOTA)-modified CD11b-specific monoclonal antibody (mAb) (clone M1/70), we were able to label *in vitro* generated polymorphonuclear (PMN-) and monocytic (M-) MDSCs for positron emission tomography (PET) imaging. Radiolabeled PMN- and M-MDSCs ([^64^Cu]PMN-MDSCs and [^64^Cu]M-MDSCs, respectively) were then adoptively transferred into primary and metastatic MMTV-PyMT-derived (PyMT-) breast cancer- and B16F10 melanoma-bearing experimental animals, and static PET and anatomical magnetic resonance (MR) images were acquired 3, 24 and 48 h post cell injection. **Results:** The internalization of the [^64^Cu]NOTA-mAb-CD11b-complex was completed within 3 h, providing moderately stable radiolabeling with little detrimental effect on cell viability and function as determined by Annexin-V staining and T cell suppression in flow cytometric assays. Further, we could non-invasively and quantitatively monitor the migration and tumor homing of both [^64^Cu]NOTA-αCD11b-mAb-labeled PMN- and M-MDSCs in mouse models of primary and metastatic breast cancer and melanoma by PET. We were able to visualize and quantify an increased migration of adoptively transferred [^64^Cu]M-MDSCs than [^64^Cu]PMN-MDSCs to primary breast cancer lesions. The frequency of endogenous MDSCs in the PyMT breast cancer and B16F10 melanoma model correlated to the uptake values of adoptively transferred MDSCs with higher frequencies of PMN- and M-MDSCs in the more aggressive B16F10 melanoma tumors. Moreover, aggressively growing melanomas and melanoma-metastatic lesions recruited higher percentages of both [^64^Cu]PMN- and [^64^Cu]M-MDSCs than primary and metastatic breast cancer lesions as early as 24 h post adoptive MDSC transfer, indicating an overall stronger recruitment of cancer-promoting immunosuppressive MDSCs. **Conclusion:** Targeting of the cell surface integrin CD11b with a radioactive mAb is feasible for labeling of murine MDSCs for PET imaging. Fast internalization of the [^64^Cu]NOTA-αCD11b-mAb provides presumably enhanced stability while cell viability and functionality was not significantly affected. Moreover, utilization of the CD11b-specific mAb allows for straightforward adaptation of the labeling approach for *in vivo* molecular imaging of other myeloid cells of interest in cancer therapy, including monocytes, macrophages or neutrophils.

## Introduction

While cancer treatment options are constantly being improved, metastatic disease is still often deemed incurable, accounting for approximately 90% of cancer-related deaths 1-3. Due to their potent immunosuppressive capacity, and apparent involvement in cancer progression and metastasis, myeloid-derived suppressor cells (MDSCs) have moved into the focus of cancer research. MDSCs are considered aberrantly activated immature cells of the myeloid compartment, whose expansion is mediated primarily by granulocyte-macrophage colony stimulation factor (GM-CSF), macrophage CSF (M-CSF) and granulocyte CSF (G-CSF), amongst other tumor-secreted growth factors 4, and likely activated by many different pro-inflammatory stimuli, such as interleukin 6 (IL-6), IL-1β, tumor necrosis factor (TNF) or interferon γ (IFN-γ) 5. MDSCs are currently divided into two phenotypically and functionally different main subpopulations in mouse and three predominant populations in man: PMN-MDSCs are described as CD11b^+^Ly6C^int^Ly6G^+^ and M-MDSCs as CD11b^+^Ly6C^high^Ly6G^-^ in mouse while, in man, PMN-MDSCs are characterized as CD11b^+^CD14^-^CD33^+^CD66^+^HLA-DR^-/low^ and M-MDSCs as CD11b^+^CD14^+^CD33^+^HLA-DR^-/low^. The third main population, described only in man, are lineage marker negative HLA-DR^-^CD33^+^ early-stage MDSCs (eMDSCs) lacking the expression of CD14/CD15 6. Once actively recruited, MDSCs in the tumor microenvironment (TME) interfere with tumor rejection by dampening the host immune response against the tumor cells 5, 7. Utilizing a plethora of different mechanisms, including the inhibition of T cell recruitment, the deprivation of nutrients compulsory for T cell proliferation in the TME or the secretion of immunosuppressive transforming growth factor β (TGF-β), MDSCs inhibit T cell and NK cell functions essential for cancer cell rejection. The MDSC-mediated recruitment of regulatory T cells further contributes to immunosuppression in the TME 5, 7. Beside their involvement in tumor progression, MDSCs participate in the orchestration of pre-metastatic niche formation in secondary organs 8, 9.

However, still little is known about the dynamics of MDSC migration *in vivo*. Over the last years, non-invasive imaging techniques such as positron emission tomography (PET) have proven to be sensitive tools to quantitatively monitor cell migration and homing dynamics *in vivo* in different areas of research, using either indirect or direct cell labeling methods 10, 11. Indirect cell labeling for PET imaging requires the introduction of an imaging reporter gene in the cell type of interest, such as the herpes simplex virus-1 thymidine kinase (HSV1-tk) with high substrate specificity to the radioactive tracers 2'-deoxy-2'-[^18^F]fluoro-5-ethyl-1-β-D-arabinofuranosyl-uracil ([^18^F]FEAU) or 9-(4-[^18^F]fluoro-3-[hydroxymethyl]butyl)guanine ([^18^F]-FHBG) 12, 13. Direct cell labeling can be readily performed *in vitro*, e.g. with lipophilic imaging probes or radiolabeled antibodies 10, 11. Herein, the PET imaging time window is limited by radionuclide half-life time. Imaging time windows of up to 48 h are enabled by the use of the lipophilic PET probe [^64^Cu]pyruvaldehyde-bis(N4-methylthiosemicarbazone) ([^64^Cu]PTSM), for example in context of inflammatory disease 10 or regenerative medicine 14. Radioactively labeled monoclonal antibodies (mAbs) or engineered antibody fragments are the most often used imaging reporters for the direct, *in situ* labeling and monitoring of endogenous immune cells in cancer 15-17.

As MDSCs expand *in vivo* from two different hematopoietic precursor cells and share cell surface markers with myeloid cells such as monocytes, macrophages and neutrophils, specific *in situ* labeling poses difficulties 18, 19. In previous work, we have labeled murine PMN-MDSCs with the fluorescent dye formulation DiD to follow their migration in primary and metastatic PyMT breast cancer-bearing mice via optical imaging (OI) 20. Due to the methodological limitations of tissue penetration and spatial resolution of OI, we have now chosen to adapt our recently established antibody-receptor targeting approach for PET imaging towards MDSCs. With this method, we could previously radiolabel murine CD4^+^ T helper cells efficiently and reliably by targeting the T cell receptor with a radioactively labeled mAb 11. In comparison to the unspecific, passive uptake of [^64^Cu]PTSM, active internalization of the receptor-antibody-complex provided higher stability of the radiolabel with simultaneously less detrimental effects on cell viability and function 11, 21. We have now successfully transferred this approach to PMN- and M-MDSCs* in vitro* generated from bone marrow progenitor cells using CD11b (integrin α_M_) as target for radiolabeling. As α_M_β_2_ heterodimer with the common integrin β_2_, CD11b is implicated in adhesion of neutrophils and monocytes to activated endothelium as well as in phagocytosis by recognition of inactivated complement components 22. Being expressed widely on both murine and human MDSC subpopulations, the cell surface-bound CD11b poses an excellent target for MDSC radiolabeling. Using a ^64^Cu-modified CD11b-specific mAb tagged with 1,4,7-triazacyclononane-triacetic acid (NOTA) as chelator, we were able to radiolabel both MDSC subpopulations with little effect on cell viability and function to reveal the kinetic of specific homing to the primary and metastatic TME in different cancer types. Sequentially, we followed the migration and tumor homing of both [^64^Cu]NOTA-αCD11b-mAb-labeled PMN- and M-MDSCs ([^64^Cu]PMN- and [^64^Cu]M-MDSCs, respectively) in mouse models of primary and metastatic PyMT breast cancer and B16F10 melanoma. Moreover, the use of the common cell surface marker CD11b for radiolabeling enables straightforward translation of this imaging approach to other CD11b^+^ cells, such as neutrophils, monocytes and macrophages.

## Methods

### Mice

C57BL/6N mice were purchased from Charles River Laboratories (Sulzfeld, Germany), C57BL/6-Tg(TcraTcrb)1100Mjb/Crl (OT-1) mice were bread in the service facility for transgenic animals at the University Hospital Tübingen. Mice were maintained under specific pathogen free conditions in individual ventilated cages with standard rodent pellet food and water ad libitum. Female C57BL/6N mice between 8-12 weeks of age were used for all experiments. All animal procedures were conducted in accordance with German federal regulations on the use and care of experimental animals, and approved by the local authorities (Regierungspräsidium Tübingen).

### Cell lines

All cell culture supplies were obtained from Merck Millipore (Biochrom, Merck Millipore, Burlington, Massachusetts, United States). The generation and maintenance of PyMT and luciferase positive (luc)-PyMT mammary tumor cell lines were previously described 9, 23. The B16F10-Luc2 melanoma cell line was purchased from Perkin Elmer (Waltham, Massachusetts, United States) and maintained in RPMI 1640 supplemented with 10 % heat-inactivated fetal calf serum (FCS). Anti-mouse αCD11b-mAb-producing M1/70 hybridoma cells (American Type Culture Collection, ATCC, Manassas, Virginia, United States) were cultured in DMEM containing 10 % heat-inactivated FCS, 1 mM sodium pyruvate, 1 % MEM-amino acids, 10 mM HEPES buffer, 100 U/mL Penicillin/Streptomycin and 0.05 mM 2-mercaptoethanol (Sigma Aldrich, St. Louis, Missouri, United States) (complete DMEM medium). All cell lines were maintained at 37 °C and 7.5 % CO_2_ in a humidified incubator.

### MDSC generation

The *in vitro* generation of bone marrow-derived MDSCs was adapted from Marigo *et al.* 24. Briefly, bone marrow cells were isolated from femurae and tibiae of female C57BL/6 mice and kept overnight on UV-irradiated or sterile petri dishes in 5 mL RPMI 1640 medium supplemented with 10 % heat-inactivated FCS, 1 mM sodium pyruvate, 1 % MEM-amino acids, 10 mM HEPES buffer, 100 U/mL Penicillin/Streptomycin and 0.05 mM 2-mercaptoethanol (complete RPMI medium). On day 1 of culture, cells were transferred to suspension cell culture plates (Sarstedt, Nümbrecht, Germany) in 10 mL complete RPMI medium supplemented with 40 ng/mL GM-CSF and 40 ng/mL IL-6 (Peprotech, Hamburg, Germany). Fresh cytokines were added on day 3 and 5 of culture, medium was changed when necessary. On day 6, the expanded PMN-MDSCs and M-MDSCs were separated according to Ly6G and Gr-1 expression by magnetic cell sorting (Miltenyi Biosciences, Bergisch Gladbach, Germany) following the manufacturer's instructions and analyzed by flow cytometry after staining with V500-αCD45.2 (clone: 104, BD Biosciences, Franklin Lakes, New Jersey, United States), APC-eFluor780-αCD11b (clone: 1A8, eBioscience, Thermo Fisher Scientific, Waltham, Massachusetts, United Stated), APC-αLy6C (clone: HK1.4, BioLegend, San Diego, California, United States) and PE-αLy6G (clone: 1A8, BD Biosciences) antibodies.

### Primary tumor and metastasis models

Primary PyMT breast cancer tumors were induced by orthotopic injection of 5x10^5^ PyMT cells in 25 µL saline into the left 4^th^ mammary fat pad. PyMT breast cancer metastases were induced by *intracardiac* (*i.c.*) injection of 2.5x10^5^ luc-PyMT cells in 100 µL saline. PyMT breast cancer primary tumors and PyMT metastases were allowed to grow for 21 days. B16F10 primary melanomas were induced by *intracutaneous* injection of 2.5x10^5^ B16F10-Luc2 cells in 25 µL saline, while metastases were induced by *i.c.* injection of 1x10^5^ B16F10-Luc2 cells in 100 µL saline. B16F10 primary melanoma and melanoma metastases were allowed to grow for 14 days. Tumor size was monitored using calipers while metastatic growth of melanomas was monitored by bioluminescence OI. All inoculation procedures were performed under 1.5 % isoflurane anesthesia (flow rate 0.8 L/min) and experimental animals received analgesics before the injections and until 3 days after the *i.c.* injection.

### CD11b mAb isolation, NOTA conjugation and radiolabeling

The rat anti-mouse αCD11b mAb (isotype: IgG_2b_) was purified from the M1/70 hybridoma cell line (ATCC) culture supernatants via affinity chromatography using HiTrap protein G columns (GE Healthcare, Little Chalfont, United Kingdom) with 1 mL column volume according to the manufacturer's instructions. The mAb concentration was determined via UV/Vis spectroscopy using a NanoDrop1000 photometer (Thermo Fisher Scientific) and adjusted to 8 mg/mL in PBS using 100 kDa Amicon Ultra-15 Centrifugal Filter Units (Merck Millipore). To eliminate trace metal contaminations, the mAb solution was incubated first with 5 µL 0.5 M EDTA (pH 7) and then washed three times in 0.1 M HEPES buffer (pH 7.5) treated with 1.2 g of Chelex 100 (Sigma Aldrich). NOTA-*N*-hydroxysuccinimide ester (NOTA-NHS, Macrocyclics, Plano, Texas, United States) was dissolved in Rotipuran Ultrapure water (Carl Roth, Karlsruhe, Germany) at a concentration of 10 mg/mL, and the conjugation reaction between the mAb and the NOTA-NHS ester was performed in a 55-fold molar excess over night at 4 °C. The NOTA-αCD11b-mAb was washed 7 times in sterile Chelex-treated 0.25 M sodium acetate (pH 6) and concentrated to 2-6 mg/mL using 100 kDa Amicon Ultra-15 Centrifugal Filter Units (Merck Millipore).

^64^Cu was produced as previously described 11, 21 and buffered to pH 5-6 with 0.5 M ammonium acetate for antibody radiolabeling. The NOTA-αCD11b-mAb was incubated with [^64^Cu]CuCl_2_ in a 2:1 ratio MBq:µg protein for 60 min at 42 °C and the reaction was quenched with 1 µL diethylenetriaminepentaacetic acid (20 mg/mL). Radiochemical purity was assessed by instant thin layer chromatography on silica gel with 0.1 M citrate (pH 5.0) as running buffer.

### Cell labeling using [^64^Cu]NOTA-αCD11b-mAb

Isolated PMN- and M-MDSCs were suspended at 2x10^6^ cells/mL in complete RPMI medium supplemented with 40 ng/mL GM-CSF and 40 ng/mL IL-6 (Peprotech). For radiolabeling, 1x10^6^ PMN- and M-MDSCs were dispensed into low adherence 48-well plates (Sarstedt). Subsequently, 0.74 MBq corresponding to 1.6 µg [^64^Cu]NOTA-αCD11b-mAb in 20 µL PBS were added to each well and incubated for 30 min at 37 °C. Cells were then washed twice and suspended in either PBS or complete medium, and the cell count was adjusted for adoptive transfer into tumor- or metastases-bearing mice or further *in vitro* investigation.

### [^64^Cu]NOTA-αCD11b-mAb uptake and efflux

The uptake of the [^64^Cu]NOTA-αCD11b-mAb into PMN- and M-MDSCs was determined by γ-counting (Perkin Elmer). Therefore, 1x10^5^ PMN- or M-MDSCs in 1 mL complete medium were transferred into γ-counting tubes directly after radiolabeling. The labeling stability and efflux of [^64^Cu]NOTA-αCD11b-mAb was determined directly, and 5, 24 and 48 h after initial radiolabeling by measuring radiolabeled [^64^Cu]PMN- and [^64^Cu]M-MDSCs and supernatants separately.

### Viability of [^64^Cu]NOTA-αCD11b-mAb-labeled MDSCs

To assess the effect of the [^64^Cu]NOTA-αCD11b-mAb labeling on the viability of PMN- and M-MDSCs, radiolabeled [^64^Cu]PMN- and [^64^Cu]M-MDSCs and unlabeled controls were stained with 7-aminoactinomycin D (7-AAD) and PE-Annexin V 3 and 48 h post radiolabeling using the PE Annexin V Apoptosis Detection Kit I (BD Biosciences). Staining was performed according to the manufacturer's instruction and samples were analyzed on a BD LSRFortessa flow cytometer (BD Biosciences). Data analysis was performed using the FlowJo software Version 10 (Tree Star, Inc., Ashland, Oregon, United States).

### Immunosuppression assay

The capacity of naïve *in vitro* expanded, αCD11b-mAb-labeled PMN- and M-MDSCs (CD11b-PMN and CD11b-M-MDSCs, respectively) and [^64^Cu]PMN- and [^64^Cu]M-MDSCs to suppress T cell proliferation was evaluated in a carboxyfluorescein succinimidyl ester (CFSE)-dilution assay. MDSCs were labeled with αCD11b-mAb or [^64^Cu]NOTA-αCD11b-mAb as described above. CD8^+^ T cells were isolated from the spleen and extraperitoneal lymph nodes of C57BL/6-Tg(TcraTcrb)1100Mjb/Crl (OT-1) mice using a T cell isolation kit (Miltenyi Biosciences). Freshly isolated OT-1 CD8^+^ T cells were stained with carboxyfluorescein diacetate succinimidyl ester at a final concentration of 1.25 µM in PBS (Cell trace Kit, Life Technologies, Thermo Fisher Scientific) and T cell proliferation was stimulated with 50 U IL-2 (Novartis, Basel, Switzerland), 8 µg/mL SIINFEKL peptide (EMC Microcollections, Tübingen, Germany) and the T Cell Activation and Expansion Kit (Miltenyi Biosciences) according to the manufacturer's instructions. CFSE-labeled OT-1 T cells and naïve *in vitro* expanded, CD11b-PMN- and CD11b-M-MDSCs or [^64^Cu]PMN- and [^64^Cu]M-MDSCs were incubated in different cell to cell ratios for 72 h in a 96-well plate. Flow cytometric analysis was performed on the BD LSRFortessa (BD Biosciences). The percentage of T cell proliferation for each condition was assessed according to CFSE-dilution in comparison to T cells alone using the FlowJo software (Tree Star).

### CD11b blocking

To assess the availability of CD11b binding sites on MDSCs after prelabeling with 1.6 µg αCD11b-mAb (clone M1/70), PMN- and M-MDSCs were stained for 30 min at 4 °C with Cy5-modified αCD11b-mAb (clone M1/70) 3, 24 and 48 h post initial prelabeling. Unlabeled PMN- and M-MDSCs served as control. Flow cytometric analysis was performed on a BD LSRII flow cytometer (BD Biosciences). Data was analyzed with FlowJo (TreeStar).

### Fluorescence microscopy

To visualize internalization of the anti-mouse Cy3-αCD11b-mAb, PMN- and M-MDSC were labeled with Cy3-αCD11b-mAb as described, cell samples were fixed in 2 % formalin, pipetted onto a microscopy slide and analyzed directly after preparation.

Staining for phosphorylated H2A.X histones as an indication for DNA-double strand breaks was performed after MDSC fixation in 2 % PBS-buffered formalin followed by two washing steps in Rotipuran Ultrapure water (Carl Roth) and 10 min heat-fixation onto microscopy slides. MDSC cell samples were permeabilized with 0.3 % Triton X-100 in 10 % donkey serum for 30 min. After washing, microscopy slides were incubated in anti-phosphorylated H2A.X antibody (1:200, Abcam) for 1 h, washed and stained with donkey anti-rabbit F(ab')_2_-Cy3 (1:500, Dianova) and donkey anti-rat F(ab')_2_-Alexa 488 (1:500, Dianova) for 1 h. Staining with DAPI (1:10000, Sigma Aldrich) was used for the visualization of cell nuclei.

For the *ex vivo* validation of MDSC migration to the primary PyMT- and B16F10 melanoma tumors, paraffin-embedded tumor tissue sections were deparaffinized, rehydrated, and incubated in citrate buffer pH 6.0 for 2 min in a pressure cooker for antigen recovery before cooling in deionized water for 10 min at room temperature. After washing, tumor tissue sections were blocked in donkey serum (1:20 dilution, 30 min) and incubated with rabbit anti-Ki-67 (1:100, Abcam, Cambridge, United Kingdom) for 1 h as a marker for proliferating tumor cells. Secondary antibody staining was performed for 1 h with anti-rabbit Alexa 647 (1:500, Dianova, Hamburg, Germany) and anti-rat-Cy3 (1:500, Dianova) to identify adoptively transferred MDSCs labeled with the rat αCD11b-mAb. Then, cell nucleus staining with Yopro nuclear dye (Molecular Probes, Thermo Fisher Scientific) was performed. All images were acquired on the LSM 800 microscope (Zeiss, Oberkochen, Germany) operated under the Zen software (Version 2.3).

### Flow cytometric profiling for endogenous MDSCs

To assess the frequency of endogenous PMN- and M-MDSCs, PyMT breast cancer and B16F10 melanoma tumors were inoculated as described, isolated and cut into small pieces (approximately 1.5-2 mm in height and width). Tumor tissue was then digested with 2 mg/mL Collagenase Type IV (Sigma Aldrich) in DMEM supplemented with 5 % FCS and 10 mM HEPES buffer for 40 min at 37 °C. Then, the tissue was washed first through a 70 µm cell strainer, then through a 40 µm cell strainer. Remaining erythrocytes were lysed with 3 mL ACK lysing buffer (Lonza, Basel, Switzerland) for 4 min at room temperature. The tumor cell suspension was then pipetted into a 5 mL polystyrene tube via a 40 µm cell strainer snap cap (Corning, New York, United States), counted and 5x10^6^ cells were used for antibody staining. Single cell suspensions were stained with BV510-αCD45 (clone: 30-F11), AF700-αB220 (clone: RA3-6B2), BV421-αLy6C (clone: HK1.4), BV605-αCD11b (clone: M1/70), BV711-αLy6G (clone: 1A8), BV785-αCD11c (clone: N418), FITC-αCD3 (clone: 500A2), PerCP-αCD4 (clone: RM4-5), BV650-αCD8a (clone: 53-6.7), PE/Cy7-αI-A/I-E (clone: M5/114.15.2) and PE-αF4/80 (clone: BM8) for 30 min at 4 °C, washed three times in PBS, fixed in 0.5 % formalin and analyzed on the BD LSRFortessa flow cytometer (BD Biosciences).

### G-CSF ELISA

Plasma levels of G-CSF in PyMT breast cancer- and B16F10 melanoma-bearing mice were determined with a standard ELISA (R&D Systems, Minneapolis, Minnesota, United States) according to the manufacturer's instructions. Therefore, retro-orbital blood was sampled from PyMT breast cancer- and B16F10 melanoma-bearing mice directly after CO_2_ asphyxiation into an EDTA-containing micro tube (Sarstedt), cellular blood components were separated via centrifugation and the resulting plasma was stored at -80 °C until use.

### Bioluminescence OI

Bioluminescence OI measurements to monitor metastatic growth of PyMT breast cancer- or B16F10 melanoma-metastatic lesions were performed with the IVIS Spectrum OI system (Perkin Elmer) on shaved and depilated experimental animals. Experimental animals were anesthetized with 1.5 % isoflurane in oxygen, injected intraperitoneally with 200 μL Luciferin (XenoLight, D-Luciferin - K+ salt, Perkin Elmer) with a final dose of 150 mg/kg bodyweight and imaged 2 min after injection to allow Luciferin to distribute. Bioluminescence images were acquired with open emission filter, medium binning and f-stop 1 in a 14x14 cm FOV as images sequences with exposure times of 10, 30, 60 and 120 sec. *Ex vivo* bioluminescence images were acquired directly after organ isolation on non-reflective black paper with the same settings as described.

### *In vivo* imaging using PET/MR

Tumor- or metastases-bearing experimental animals and naïve littermate controls were anesthetized with 1.5 % isoflurane in oxygen with a flow rate of 0.8 L/min in a temperature-controlled anesthesia box. For the *intravenous* (*i.v.*) adoptive cell transfer, a catheter was inserted into the tail vein directly before injection of either 2x10^6^ [^64^Cu]PMN-MDSCs or [^64^Cu]M-MDSCs corresponding to approximately 0.028-0.037 MBq. Static 20 min emission PET scans were acquired 3, 24 and 48 h post adoptive cell transfer on a dedicated small-animal Inveon microPET scanner (Siemens Healthineers, Erlangen, Germany), followed by T2-weighted anatomical magnetic resonance (MR) scans using a TurboRARE protocol (TR = 1800 ms; TE = 90.51 ms; field of view 76.8x34.8x22.8 mm³, image size 256x116x76) with a rat whole-body volume coil (inner diameter: 86 mm) on the 7 T BioSpec 70/30 MR scanner (Bruker, Ettlingen, Germany).

### PET image reconstruction, data analysis and statistics

PET images were reconstructed as previously described 11 in Inveon Acquisition Workplace 1.5.0.28 (Siemens Healthineers). Briefly, static histogram files were constructed from list-mode data and images were reconstructed by applying an ordered subset expectation maximization algorithm in two dimensions (Fourier rebinning, OSEM2D, 16 subsets, 4 iterations, pixel size 128x128, matrix 0.79x0.79 mm²). Attenuation correction was not performed. PET images were corrected for radioactive decay, normalized to the amount of injected activity and fused with the anatomical MR images with the help of glass capillaries containing [^64^Cu]NOTA-αCD11b-mAb solution in Inveon Research Workplace (Siemens Healthineers). Volumes of interest (VOI) were drawn on the lung, liver and spleen on the anatomical MR image, cell uptake in primary tumors and metastases was quantified by drawing VOIs on hot spots on the PET images co-registered to the MR images for anatomical guidance. The percentage of injected dose per cubic centimeter (%ID/cm³) values were calculated as follows: mean activity in VOI/(injected activity*10^6^)/100.

### *Ex vivo* biodistribution analysis

To validate the *in vivo* PET data, the biodistribution of [^64^Cu]PMN-MDSCs and [^64^Cu]M-MDSCs was analyzed by γ-counting* ex vivo* after the 48 h imaging time point. Retro-orbital blood was sampled directly after the animal was sacrificed by CO_2_ asphyxiation. For the primary tumor models, the tumor, lungs, liver, spleen, kidneys, the left femur and tibia and muscle tissue were harvested for biodistribution analysis.

For the metastasis models, tissue margins between healthy and cancerous tissue were determined by *ex vivo* bioluminescence OI and the cancerous tissue was separated from the healthy organ as far as technically possible. In this way, metastases were separated from the heart, lungs, liver, spleen, kidneys, adrenal glands, ovaries, femurae and tibiae and subjected to γ-counting separately from the healthy tissue.

A standard solution with a known amount of radioactivity served as reference to calculate the percentage of injected dose per gram of tissue (%ID/g). The tubes containing the standard solution and the organs were measured in the Wizard2 automated γ-counter (Perkin Elmer) at an energy window of 350 to 650 keV. The resulting decay corrected counts per minute were then first normalized to the injected dose with the help of the standard solution and to the respective weight of the organ to obtain %ID/g.

### Statistics

Comparative %ID/cm³ values of experimental groups are given as mean ± SEM. For statistical analysis, two-tailed Student's t-test or Dunnett's Multiple Comparison Test were applied. Resulting p-values <0.05 were considered significant (*).

## Results

### Characterization of bone marrow-derived MDSCs

**Figure 1:**
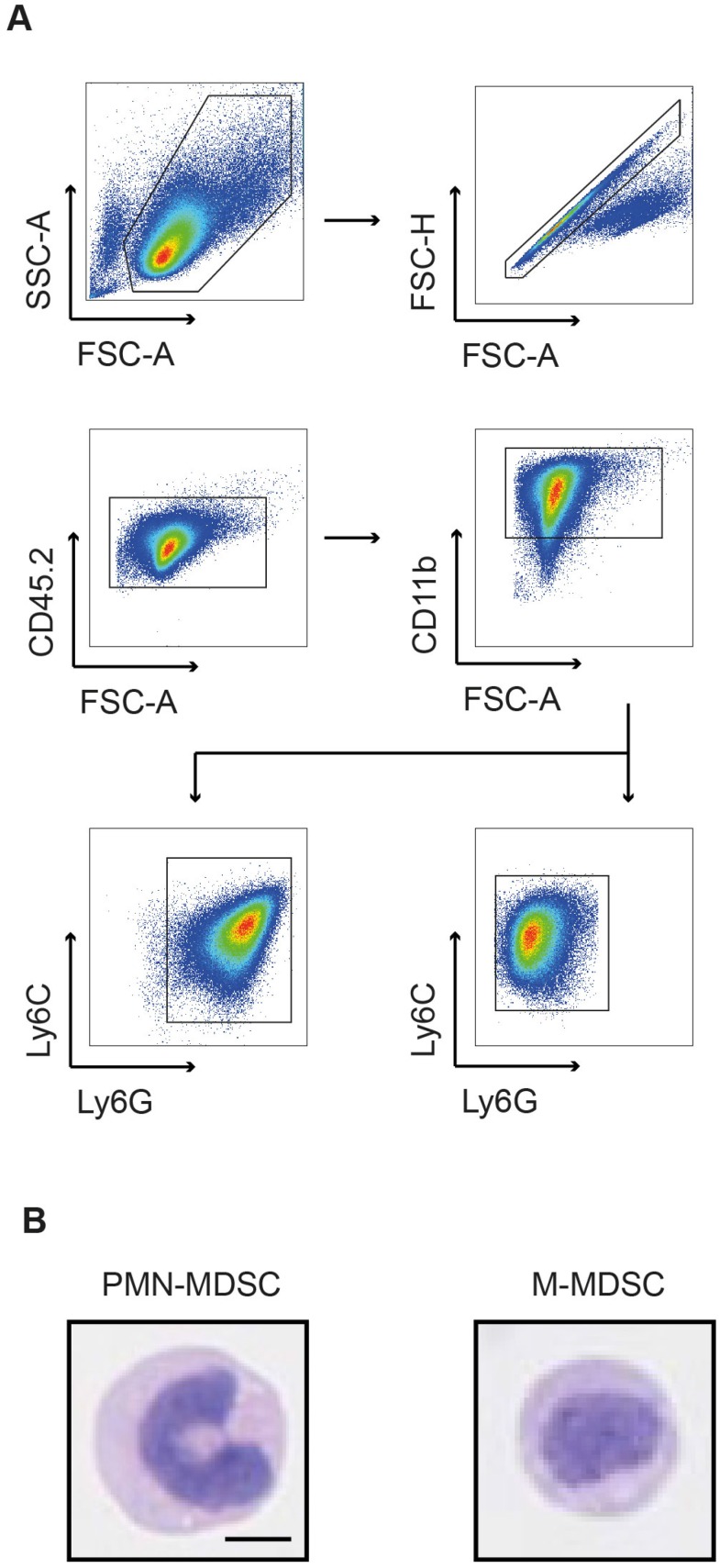
** Flow cytometric and morphologic characterization of bone marrow-derived MDSCs.** (**A**) Flow cytometric analysis of bone-marrow derived MDSCs characterized PMN-MDSCs as CD11b^+^Ly6C^+^Ly6G^+^ and M-MDSCs as CD11b^+^Ly6C+Ly6G^-^. (**B**) Exemplary H&E staining of bone marrow-derived MDSCs confirmed the characteristic nuclear morphology of PMN-MDSCs (ring-like, left panel) and M-MDSCs (round, right panel). Scale bar 5 µm.

In tumor-bearing hosts, MDSC expand from progenitors in the bone marrow under the influence of growth factors and pro-inflammatory cytokines. Hence, PMN- and M-MDSCs used for cell tracking studies were expanded from bone marrow-derived progenitor cells with GM-CSF and IL-6 according to a previously published protocol 24. The resulting cells were magnetically separated and subsequently subjected to flow cytometric and morphologic characterization. The assessed phenotypes were characteristic for PMN-MDSCs and M-MDSCs, respectively (Figure 1A). PMN-MDSCs were characterized as CD11b^+^Ly6C^+^Ly6G^+^ with a ring-shaped nucleus (Figure 1B, left panel) while M-MDSCs were characterized as CD11b^+^Ly6C^+^Ly6G^-^ with a round nucleus (Figure 1B, right panel).

### Labeling of PMN- and M-MDSCs with a Cy5-conjugated CD11b-specific mAb and target internalization

**Figure 2:**
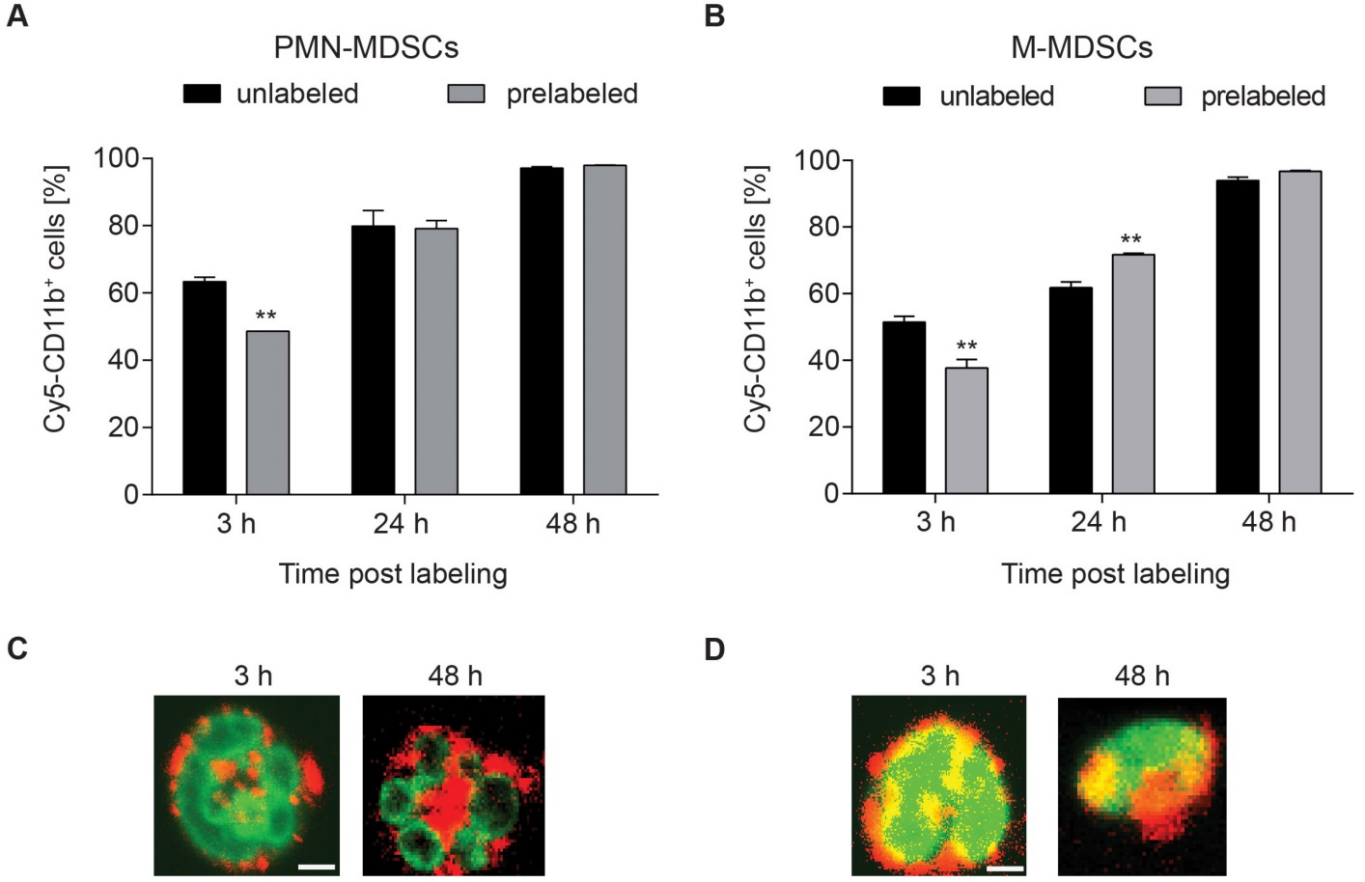
** Internalization of the αCD11b-mAb-CD11b complex and CD11b re-expression.** Flow cytometric quantification of CD11b expression by Cy5-αCD11b staining of PMN-MDSCs (**A**) and M-MDSCs (**B**) 3, 24 and 48 h after prelabeling with 1.6 µg αCD11b-mAb revealed a reduced CD11b availability at 3 h post prelabeling and CD11b re-expression after 24 h. Confocal microscopy of PMN- (**C**) and M-MDSCs (**D**) 3 h and 48 h after labeling with 1.6 µg Cy3-αCD11b proved internalization of the CD11b-antibody complex within 3 h and retention in the cells over 48 h. Green - YoPro, red - Cy3-αCD11b, scale bar: 1 µm. All values are given as mean percent of total cells ± SEM, n=3, statistics: Student's t-test with ^**^p<0.01.

The integrin CD11b was selected as target for radiolabeling of MDSCs. Being involved in adhesion and endocytotic processes of complement-bound proteins, fast internalization and re-expression of CD11b were assumed 25. To assess the amount of accessible CD11b-binding sites, we conjugated the fluorescent dye Cy5 to the CD11b-specific mAb and stained both PMN- and M-MDSC with Cy5-αCD11b-mAb at 3, 24 and 48 h after initial 30 min prelabeling with 1.6 µg αCD11b-mAb (Figure 2). CD11b-prelabeling reduced the available binding sites on both PMN- and M-MDSCs at 3 h (48.63 ± 2.16 % vs. 63.40 ± 1.28 %, **p<0.01 for PMN- and 37.67 ± 2.65 % vs. 51.43 ± 1.74 %, **p<0.01 for M-MDSCs, respectively), while CD11b re-expression was completed at 24 h post labeling (79.13 ± 2.44 % vs. 79.90 ± 4.64 % for PMN-MDSCs and 71.67 ± 0.42 % vs. 61.80 ± 1.78 %, **p<0.01 for M-MDSCs). Comparable to 24 h post prelabeling, there was no significant reduction in CD11b expression in prelabeled MDSC samples compared to unlabeled controls (97.97 ± 0.09 % vs. 97.13 ± 0.41 % for PMN-MDSCs and 96.73 ± 0.24 % vs. 93.93 ± 1.00 % for M-MDSCs) at 48 h (Figure 2A-B). The internalization of the mAb into PMN- and M-MDSCs was verified by confocal microscopy of a Cy3-modified CD11b-specific mAb directly depicting the internalization of the Cy3-αCD11b-mAb-integrin complex as early as 3 h post Cy3-αCD11b-mAb labeling of the cells (Figure 2C-D).

### *In vitro* evaluation of MDSC labeling with a ^64^Cu-radiolabeled NOTA-modified CD11b-mAb

**Figure 3:**
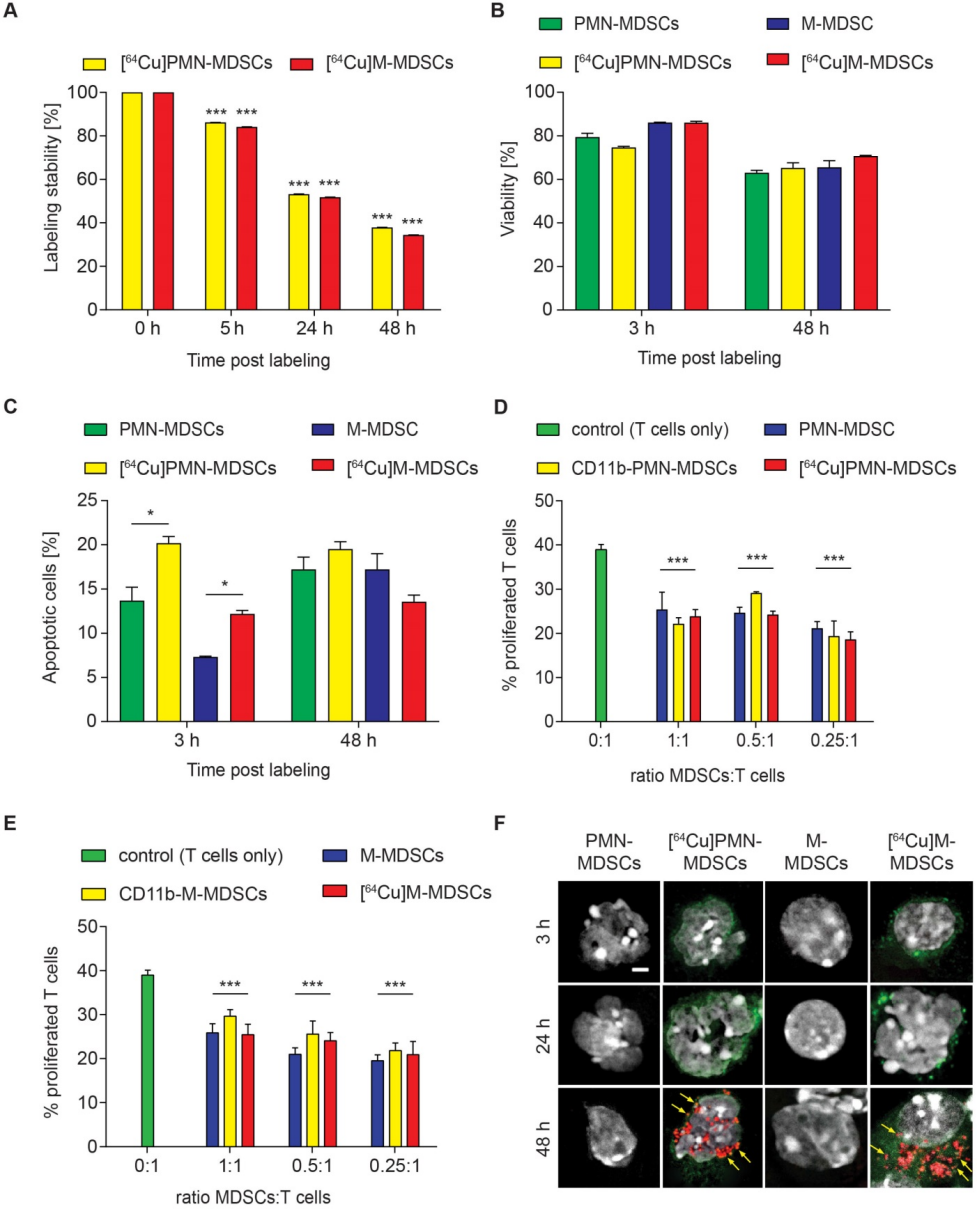
** Evaluation of [^64^Cu]NOTA-αCD11b-mAb labeling effects on PMN- and M-MDSCs.** (**A**) *In vitro* evaluation of [^64^Cu]NOTA-αCD11b-mAb labeling stability on PMN-MDSCs and M-MDSCs 5, 24 and 48 h after radiolabeling measured by γ-counting reveals only mediocre stability of the radiolabel at 48 h after radiolabeling. Data is normalized to the 0 h time point measured directly after radiolabeling as initial activity in PMN- and M-MDSCs, respectively (mean ± SEM in percent, n=9, statistics: Dunnett's Multiple Comparison Test, ***p<0.001). Flow cytometric analysis of viability (**B**) and apoptosis induction (**C**) of PMN- and M-MDSCs after labeling with the [^64^Cu]NOTA-αCD11b-mAb revealed reduced viability and significantly enhanced apoptosis induction in [^64^Cu]PMN- and [^64^Cu]M-MDSCs at 3 h after radiolabeling compared to unlabeled controls (mean ± SEM in percent, n=3) while the effect of radiolabeling was not as pronounced at 48 h after radiolabeling. Immunosuppression assay with *in vitro* expanded naïve PMN-(**D**)/M-MDSCs (**E**), CD11b-PMN-/M-MDSCs and [^64^Cu]PMN- and [^64^Cu]M-MDSCs demonstrated no significant loss in functionality as measured by capacity to inhibit antigen-induced proliferation of CFSE-labeled OT-1 CD8^+^ T cells after radiolabeling (mean ± SEM in percent, n=4, statistics: Dunnett's Multiple Comparison Test, ***p<0.001). Representative images of immunofluorescence staining for phosphorylated H2A.X histones as early markers for DNA damage (**F**) at 3, 24 and 48 h after radiolabeling with [^64^Cu]NOTA-αCD11b-mAb revealed DNA damage only at 48 h post radiolabeling in [^64^Cu]PMN- and [^64^Cu]M-MDSCs while no signs for DNA damage could be detected in unlabeled controls. Red - phosphorylated H2A.X, green - αCD11b mAb, white - DAPI nuclear dye, scale bar: 2 µm. Yellow arrows indicate sites of phosphorylated H2A.X histones.

According to our previously published evaluation on the amount of radioactivity dosage for murine CD4^+^ T cell labeling 11, PMN- and M-MDSCs were radiolabeled with 0.74 MBq [^64^Cu]NOTA-αCD11b-mAb/10^6^ cells (corresponding to 1.6 µg mAb) for 30 min at 37 °C. This approach resulted in a high stability of radioactivity on both [^64^Cu]PMN- and [^64^Cu]M-MDSCs at 5 h post radiolabeling (86.17 ± 0.03 % of initially measured radioactivity for [^64^Cu]PMN-MDSCs and 84.12 ± 0.02 % of initially measured radioactivity for [^64^Cu]M-MDSCs, Figure 3A) while at 24 h post radiolabeling, approximately 50 % of initially applied radioactivity was detectable in both MDSC subpopulations (53.23 ± 0.01 % for [^64^Cu]PMN-MDSCs and 51.81 ± 0.02 % for [^64^Cu]M-MDSCs). Intracellular radioactivity was further decreased at 48 h post initial radiolabeling (37.89 ± 0.01 % of initially measured radioactivity for [^64^Cu]PMN-MDSCs and 35.43 ± 0.02 % of initially measured radioactivity for [^64^Cu]M-MDSCs) (Figure 3A). Beside positron emission, ^64^Cu decays via electron capture resulting in the generation of high-energy Auger electrons that can elicit DNA damage when in close proximity to the nucleus 26. DNA damage can impair cell functionality, reduce cell viability and lead to the induction of apoptosis. Therefore, we evaluated the effect of cell labeling with 0.74 MBq [^64^Cu]NOTA-αCD11b-mAb on cell viability and apoptosis induction in PMN- and M-MDSCs. A non-significant decrease of viability of [^64^Cu]PMN-MDSCs (74.74 ± 0.50 % vs. 79.43 ± 1.78 % viable control PMN-MDSCs) and [^64^Cu]M-MDSCs (86.06 ± 0.63 % vs. 86.10 ± 0.17 % viable control M-MDSCs) was detected by 7-AAD staining at 3 h post radiolabeling (Figure 3B).

Viability of both [^64^Cu]PMN-MDSCs (65.27 ± 0.50 % vs. 63.00 ± 1.19 % viable control PMN-MDSCs) and [^64^Cu]M-MDSCs (70.70 ± 0.31 % vs. 65.53 ± 3.08 % viable control M-MDSCs) decreased further over the 48 h examination period, however, no significant effect of the radiolabel was detected (Figure 3B). The fraction of phosphatidylserine-exposing [^64^Cu]PMN-MDSCs (20.17 ± 0.77 % vs. 13.70 ± 1.53 % of unlabeled control PMN-MDSCs) and [^64^Cu]M-MDSCs (12.20 ± 0.40 % vs. 7.31 ± 0.10 % of unlabeled control M-MDSCs) increased significantly at 3 h post radiolabeling in comparison to untreated controls as detected by PE-Annexin V staining (Figure 3C). At 48 h post radiolabeling, a further increase in Annexin V-positive [^64^Cu]M-MDSCs (13.57 ± 0.75 % vs. 17.23 ± 1.77 % of unlabeled controls) and [^64^Cu]PMN-MDSCs (19.50 ± 0.85 % vs. 17.23 ± 1.37 % of unlabeled controls) could be detected while, however, no significant effect of the [^64^Cu]NOTA-αCD11b-mAb labeling was detected (Figure 3C). Since their immunosuppressive feature is considered a key function defining MDSCs, cell functionality after [^64^Cu]NOTA-αCD11b-mAb labeling was measured as capacity to suppress antigen-induced T cell proliferation. Antibody labeling with αCD11b-mAb had no statistically significant effect on the immunosuppressive capacity of PMN-MDSCs or M-MDSCs compared to unlabeled controls (Figure 3D and E, respectively). Likewise, labeling with [^64^Cu]NOTA-αCD11b-mAb did not have any significant effect on the suppressive activity of PMN- and M-MDSCs (Figure 3D and E, respectively). The phosphorylation of H2A.X family histones was assessed as an early sign for DNA damage by immunofluorescence staining. Although ^64^Cu internalization into the cells led to radiation-induced DNA double strand breaks only at 48 h post radiolabeling in both [^64^Cu]PMN- and [^64^Cu]M-MDSCs (Figure 3F), cell functionality was not overtly affected (Figure 3D-E), suggesting that this cell labeling approach was feasible for *in vivo* cell migration studies.

### Tumor homing of [^64^Cu]PMN- and [^64^Cu]M-MDSCs to primary breast cancer and melanoma

**Figure 4:**
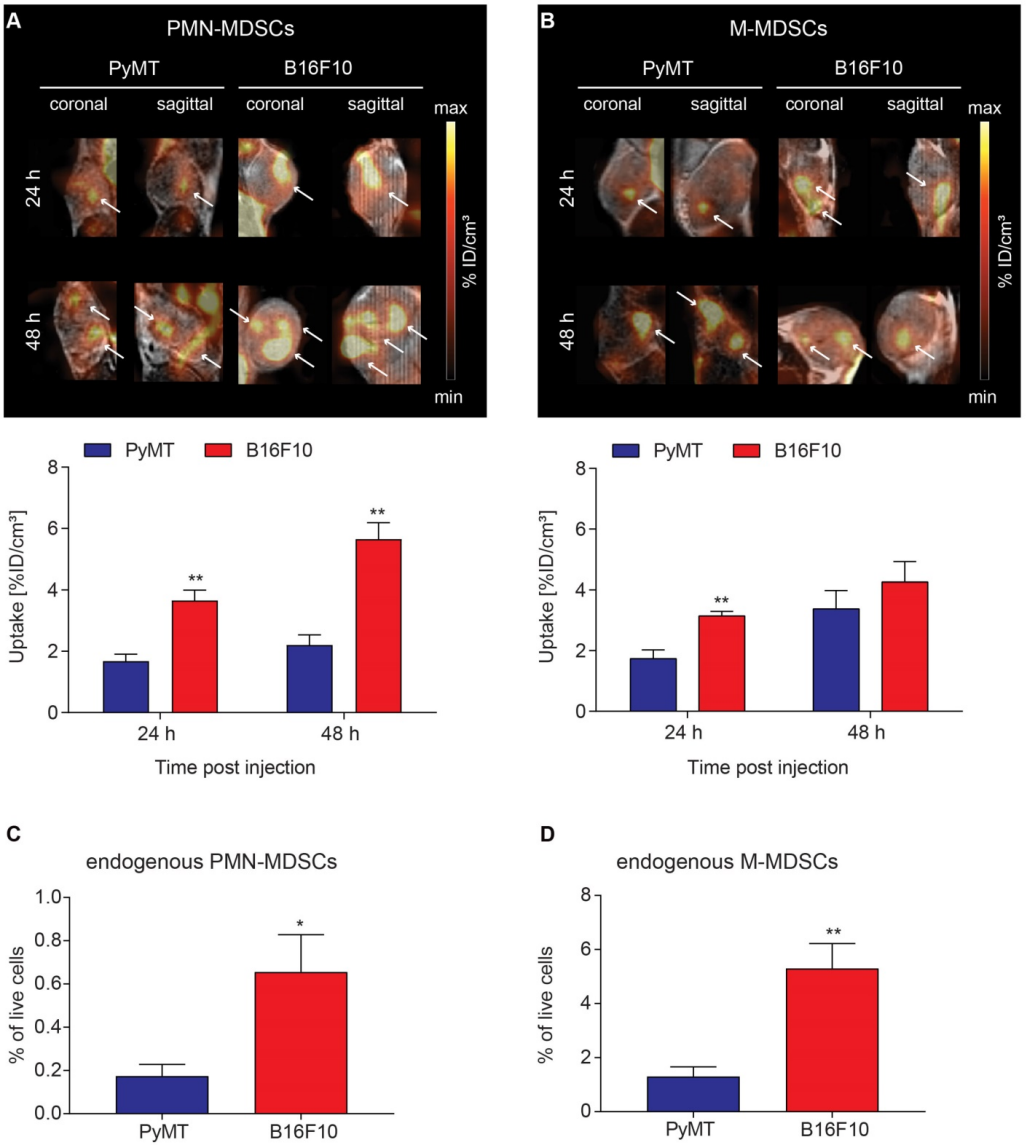
** Visualization of [^64^Cu]PMN-MDSC and [^64^Cu]M-MDSC homing to primary tumors and correlation with endogenous MDSCs.** Representative coronal and sagittal PET/MR images of (**A**) [^64^Cu]PMN-MDSCs and (**B**) [^64^Cu]M-MDSCs in primary PyMT breast tumors (left panel) and primary B16F10 melanoma (right panel). White arrows highlight hot spots of [^64^Cu]PMN- and [^64^Cu]M-MDSC uptake in the tumors revealing non-homogeneous cell distribution. Quantification of [^64^Cu]PMN-MDSC (**A**, lower panel) and [^64^Cu]M-MDSC (**B**, lower panel) migration 24 h and 48 h post injection (mean ± SEM in %ID/cm³, [^64^Cu]PMN-MDSC: n=5 for PyMT, n=6 for B16F10 and [^64^Cu]M-MDSC: n=6 for PyMT, n=5 for B16F10, statistics: Student's t-test, **p<0.01) revealed increased [^64^Cu]PMN- and [^64^Cu]M-MDSC homing to B16F10 melanomas in comparison to PyMT tumors. Correlation of uptake values of adoptively transferred [^64^Cu]PMN- and [^64^Cu]M-MDSCs to the endogenous PMN- (**C**) and M-MDSCs (**D**) in the PyMT and B16F10 tumors revealed a similar pattern of MDSCs in the tumors with higher cell frequencies of PMN- and M-MDSCs in the primary B16F10 tumors than the PyMT tumors (n=4, statistics: two-tailed Student's t-test, *p<0.05, **p<0.01).

To visualize *in vivo* MDSC migration by PET imaging, 2x10^6^ [^64^Cu]PMN- or [^64^Cu]M-MDSCs were injected *i.v.* into PyMT breast tumor- or B16F10 melanoma-bearing mice directly after radiolabeling. Static PET scans and T2-weighted anatomical MR scans were acquired 3, 24 and 48 h after MDSC transfer. As cell migration is relatively slow compared to metabolic processes, significant fractions of [^64^Cu]PMN- and [^64^Cu]M-MDSCs were detected in PyMT breast or B16F10 melanoma tumors only at 24 h after adoptive cell transfer. At 24 h after adoptive cell transfer, a significantly higher fraction of [^64^Cu]PMN-MDSCs could be located in B16F10 melanomas compared to PyMT breast tumors (3.66 ± 0.34 %ID/cm³ vs 1.67 ± 0.23 %ID/cm³, **p<0.01) (Figure 4A). Moreover, more [^64^Cu]M-MDSCs migrated into B16F10 melanomas than into PyMT breast tumors (3.14 ± 0.15 %ID/cm³ vs 1.74 ± 0.29 %ID/cm³, **p<0.01, Figure 4B). At 48 h post adoptive cell transfer, PET imaging showed a significantly higher uptake of [^64^Cu]PMN-MDSCs in B16F10 melanomas than in PyMT breast tumors (5.65 ± 0.49 %ID/cm³ vs 2.21 ± 0.30 %ID/cm³, **p<0.01, Figure 4A). No significant difference in the recruitment of [^64^Cu]M-MDSCs to both tumor types was detected 48 h post injection (Figure 4B). Compared to [^64^Cu]PMN-MDSCs, a higher fraction of [^64^Cu]M-MDSCs migrated to the PyMT breast tumors 48 h post *i.v.* injection. Interestingly, more [^64^Cu]PMN-MDSCs tended to migrate to B16F10 melanomas compared to [^64^Cu]M-MDSCs at both 24 and 48 h post injection. The differences in the uptake of MDSC subpopulations into PyMT breast cancer tumors and B16F10 melanoma indicate specific MDSC recruitment mechanisms to different tumor types. Furthermore, at all examined time points, the PET signal derived from [^64^Cu]PMN-MDSCs and [^64^Cu]M-MDSCs in both cancer types was not homogeneously distributed throughout the whole tumor but rather constituted hot spot areas (Figure 4A-B). The lungs, liver and spleen of primary PyMT breast cancer- and B16F10 melanoma-bearing mice represented further sites of [^64^Cu]PMN-MDSCs (Figure S1) and [^64^Cu]M-MDSCs (Figure S2) recruitment.

To correlate the uptake of adoptively transferred [^64^Cu]PMN- and [^64^Cu]M-MDSCs to endogenous MDSCs in the examined tumor models, PyMT breast cancer and B16F10 melanoma tumors were further examined for the frequency of endogenous PMN- (Figure 4C) and M-MDSCs (Figure 4D) by flow cytometry according to the expression of CD11b, Ly6C and Ly6G. Therefore, single cell suspensions were prepared from PyMT breast cancer and B16F10 melanoma tumors, stained and analyzed for CD11b, Ly6C and Ly6G expressing cell populations. Interestingly, B16F10 melanoma tumors showed significantly higher levels of both endogenous PMN- (0.65 ± 0.17 % vs. 0.17 ± 0.06 %, *p<0.05) and M-MDSCs (5.30 ± 0.93 % vs. 1.30 ± 0.36 %, **p<0.01) than PyMT tumors. However, the frequency of M-MDSCs (Figure 4D) was considerably higher in both PyMT breast and B16F10 melanoma tumors. As M-MDSCs were identified as CD11b^+^Ly6C^+^Ly6G^-^ cells, however, a clear and unambiguous discrimination of M-MDSCs and other tumor-resident myeloid cells was not possible.

### Recruitment of [^64^Cu]PMN- and [^64^Cu]M-MDSCs to lung metastatic lesions

Recent theories describe metastasis as a multi-step process including preceding pre-metastatic niche formation accompanied by immune evasion in secondary organs 8. The microenvironment of established metastases should therefore differ from the primary TME and, in conclusion, recruitment dynamics of MDSCs to metastases might differ from primary tumor homing. To examine a possible difference in MDSC migration kinetics to the primary and metastatic microenvironments, PyMT breast cancer and B16F10 melanoma metastases were induced by *i.c.* injection of tumor cells into the left ventricle. Metastases-bearing mice received an adoptive transfer of 2x10^6^ [^64^Cu]PMN- or [^64^Cu]M-MDSCs via *i.v.* injection and PET and MR images were acquired 3, 24 and 48 h post MDSC transfer. Bioluminescence OI images were acquired at 48 h post adoptive transfer.

**Figure 5:**
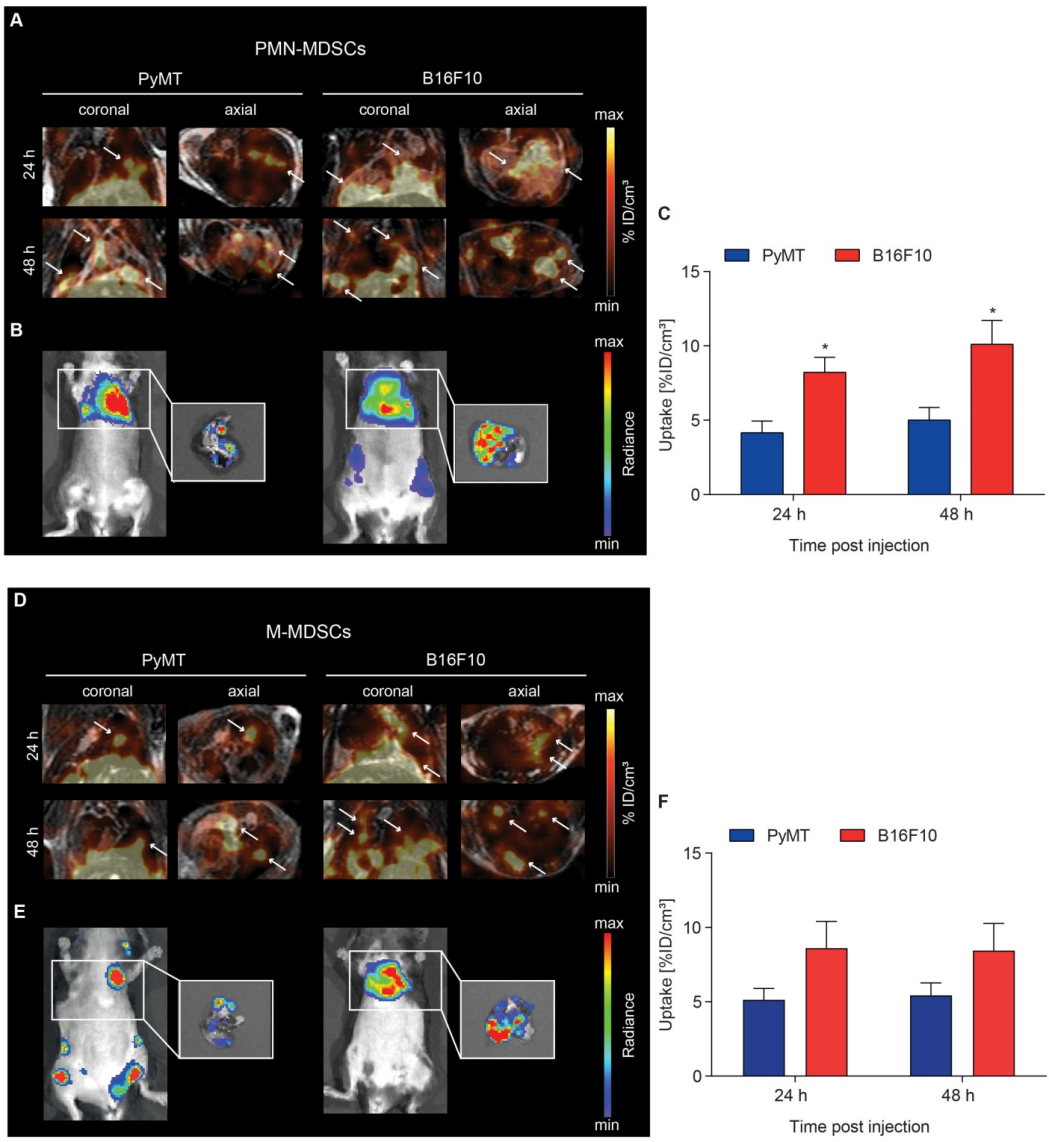
** Visualization of [^64^Cu]PMN- and [^64^Cu]M-MDSC homing to lung metastatic PyMT breast cancer and B16F10 melanoma.** Representative coronal and axial PET/MR images of [^64^Cu]PMN-MDSCs (upper panel, **A**) and [^64^Cu]M-MDSC (lower panel, **D**) in PyMT breast cancer lung metastatic lesions (left panel) and B16F10 melanoma lung metastatic lesions (right panel). White arrows highlight hot spots of [^64^Cu]PMN-MDSCs and [^64^Cu]M-MDSCs in the metastatic lungs. *In vivo* whole-body and *ex vivo* (white boxes, zoom on the isolated lungs after sacrifice) bioluminescence OI images of the metastases-bearing mice (**B** and **E**, respectively) were acquired 48 h post adoptive cell transfer. Quantification of cell migration 24 h and 48 h post injection demonstrates enhanced [^64^Cu]PMN-MDSC (**C**) and [^64^Cu]M-MDSC (**F**) homing to the B16F10 melanoma metastatic microenvironment as compared to PyMT breast cancer metastatic microenvironment (mean ± SEM in %ID/cm³, [^64^Cu]PMN-MDSC: n=7 for PyMT, n=6 for B16F10 and [^64^Cu]M-MDSC: n=6 for PyMT, n=5 for B16F10, statistics: two-tailed Student's t-test, * p<0.05).

At 24 h post adoptive transfer, MDSC recruitment to B16F10 melanoma lung metastatic lesions was significantly higher in comparison to PyMT breast cancer lung metastatic lesions for both [^64^Cu]PMN- (8.21 ± 1.02 %ID/cm³ vs 4.16 ± 0.79 %ID/cm³, ^*^p<0.05, Figure 5A and C) and [^64^Cu]M-MDSCs (8.61 ± 1.80 %ID/cm³ vs 5.13 ± 1.71 %ID/cm³, Figure 5D and F). [^64^Cu]PMN-MDSC uptake further increased at 48 h post transfer, with significantly higher cell fractions in B16F10 melanoma metastatic lesions compared to PyMT breast cancer metastatic lesions (10.10 ± 1.60 %ID/cm³ vs 5.01 ± 0.85 %ID/cm³, ^*^p<0.05, Figure 5A and C). A slight but not significant increase of [^64^Cu]M-MDSC uptake was found in both cancer metastatic lesions at 48 h post transfer (Figure 5D and F). Representative bioluminescence images of the PyMT breast cancer and B16F10 melanoma metastases-bearing animals are given in Figure 5B and E. At all examined time points, recruitment of both [^64^Cu]PMN- and [^64^Cu]M-MDSCs to B16F10 melanoma lung metastatic lesions was enhanced compared to PyMT breast cancer metastatic lesions. Furthermore, in comparison to the primary tumors, both metastatic lesions showed enhanced recruitment of both [^64^Cu]PMN and [^64^Cu]M-MDSCs, indicating differences in the capacity to recruit immunosuppressive MDSC to the respective microenvironment. Other sites of [^64^Cu]PMN-MDSC and [^64^Cu]M-MDSC recruitment in both metastasis models included the liver and spleen (Figure S3 and S4, respectively).

### *Ex vivo* validation

**Figure 6:**
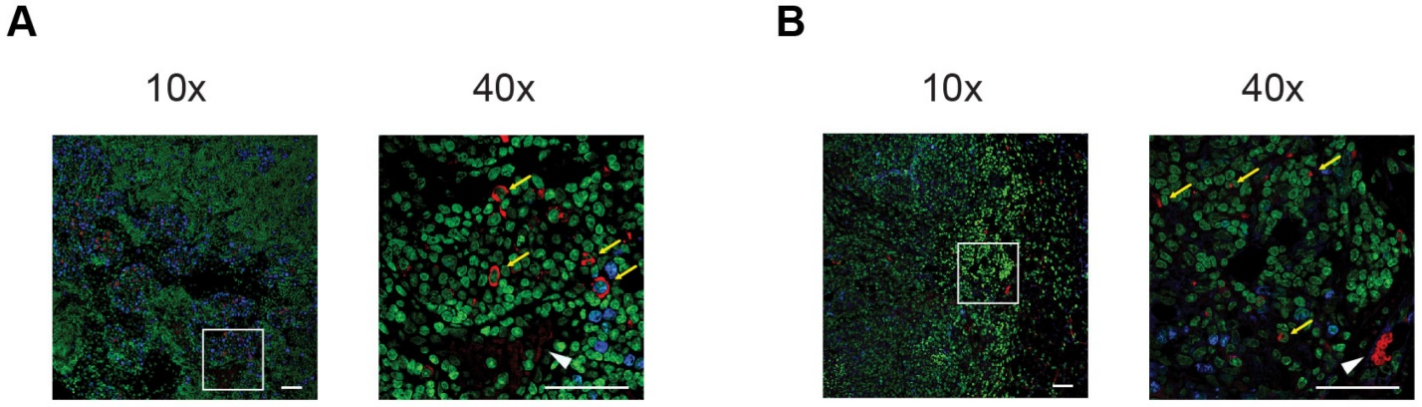
** Immunofluorescence staining confirms the *in vivo* cell trafficking data.** Representative immunofluorescence stainings of PyMT breast cancer tumors after adoptive transfer of [^64^Cu]PMN-MDSCs (**A**) or [^64^Cu]M-MDSCs (**B**), respectively, confirm that the *in vivo* signal detected as hot spot areas in PET imaging in the PyMT breast tumors is derived from adoptively transferred [^64^Cu]PMN- or [^64^Cu]M-MDSCs. Accordingly, both [^64^Cu]PMN- and [^64^Cu]M-MDSCs are not distributed homogeneously throughout the tumor sample but localize in close proximity to blood vessels. Yellow arrows indicate adoptively transferred MDSCs, white arrow head indicated blood vessel. Blue - Ki-67, green - Yopro nuclear dye, red - αCD11b mAb, scale bar 50 µm.

To confirm *in vivo* tumor homing of [^64^Cu]PMN- and [^64^Cu]M-MDSCs, experimental animals were sacrificed, primary PyMT breast tumors excised and prepared for histological staining. Due to the ambiguity of the cell surface markers for murine MDSCs, we aimed to identify adoptively transferred MDSCs by means of their antibody label for PET imaging. By staining for the rat anti-mouse αCD11b-mAb, we could validate data gathered from *in vivo* cell tracking experiments: PMN- (Figure 6A) and M-MDSCs (Figure 6B) could be identified in relative close proximity to blood vessels in the cancer mass.

## Discussion and Conclusions

By suppression of both innate and adaptive immune responses, MDSCs are key players, not only in tumor progression but also in pre-metastatic niche formation and metastasis 5, 7. In cancer patients, high percentages of MDSCs in peripheral blood are generally associated with poor prognosis 27-29. Although MDSCs are under close investigation, *in vivo* migration of MDSCs has not yet been studied extensively with non-invasive imaging. In previous work, we were able to visualize migration of *in vitro* generated, DiD-labeled PMN-MDSCs to primary PyMT breast tumors with fluorescence OI 20. In a similar study, Combes *et al.* followed the migration of MDSCs labeled with DiR, a dye closely related to DiD, in BALB/c mice bearing syngeneic 4T1 breast tumors with fluorescence OI. Herein, the authors compared *in vitro* generated MDSCs and MDSCs isolated from the bone marrow of tumor-bearing mice, however, subpopulations were not separated before adoptive transfer 30. In the second MDSC tracking study published so far, Gr1^+^ MDSCs isolated from the spleen of cervical cancer-bearing mice were labeled with superparamagnetic iron oxide particles (SPIOs) for MR imaging in subcutaneous cervical cancer-bearing mice. Adoptively transferred MDSCs were detected as signal hypointensity in the tumors 31. The detection of hypointense regions, however, constitutes a major drawback of cell tracking with SPIOs as necrotic or cystic areas in tumors might be detected as hypointense signals as well.

We here present, to our knowledge, the first MDSC tracking study employing PET as quantitative non-invasive imaging modality. We have successfully applied our previously established cell labeling protocol 11, 21 on MDSCs by use of the [^64^Cu]NOTA-αCD11b-mAb to label MDSCs *in vitro*. Radiolabeling of the MDSCs *in vitro* did not result in an unreasonably high cell loss during labeling or compromise viability of the cells. Apoptosis induction, however, was more severe in [^64^Cu]PMN-MDSCs compared to [^64^Cu]M-MDSCs at both assessed time points post initial radiolabeling. Interestingly, M-MDSCs seem to express higher levels of cell death inhibiting molecules and proteins 32, which might explain the observed differences in the fraction of apoptotic cells after radiolabeling. Internalization of the mAb-integrin-complex for intracellular labeling was faster than expected from previous studies and completed within 3 h. This might be due to the different signaling and membrane shuttling kinetics of T cell receptors and integrins. Furthermore, the mAb-integrin-complex should only be approximately half the size of the previously examined mAb-TCR-complex, which might affect the efficiency and speed of the internalization. Interestingly, overall CD11b expression increased over 48 h in both prelabeled and control MDSC samples presumably due to stimulation mediated by the cytokine supplements in the growth medium. Enhanced CD11b cell surface expression by translocation of preformed CD11b to the cell membrane was reported in neutrophils upon activation or stimulation 33, 34. Radiation, especially the decay of ^64^Cu via the emission of highly energetic Auger electrons, might induce DNA double strand breaks. Although a small fraction of both [^64^Cu]PMN- and [^64^Cu]M-MDSCs did stain positive for phosphorylated H2A.X as early sign for DNA damage at 48 h post initial radiolabeling, we did not expect pronounced effects on cell behavior during the following experiments as the observation period for cell tracking with ^64^Cu was set to 48 h only. MDSC cell functionality after radiolabeling was measured as suppressive activity against antigen-induced T cell proliferation. While the capacity to inhibit T cell proliferation was not significantly reduced in [^64^Cu]PMN- and [^64^Cu]M-MDSCs in comparison to unlabeled control MDSCs, a characteristic reduction of T cell proliferation with increasing numbers of MDSCs was not observed. Additionally, the percentage of proliferated T cells was low in the T cell only control even under stimulation with the T cell-cognate antigen. As the interplay between activated T cells and MDSCs has recently been described as pivotal for MDSC suppressive function 35, we concluded that due to the low activation state of the T cells no difference between the examined conditions could be observed. Furthermore, the stability of the radiolabel was assessed in both [^64^Cu]PMN- and [^64^Cu]M-MDSCs. Although the efflux of radioactivity from [^64^Cu]PMN- and [^64^Cu]M-MDSCs was higher than expected from previously examined murine T cells 11, the stability of the antibody-based radiolabeling was still superior to [^64^Cu]PTSM in murine T cells 11 and presumably other compounds usually used for cell labeling such as 2-deoxy-2-[^18^F]fluoroglucose 36, [^111^In]Oxine or [^99m^Tc]HMPAO 37. This discrepancy, however, emphasizes the importance of the *in vitro* evaluation of the mAb used for receptor targeting of the specific cell type of interest.

As common myeloid cell marker, CD11b can serve as a target for radiolabeling different kinds of myeloid cells including monocytes, macrophages or neutrophils. Therefore, further translation of this indirect radiolabeling approach to other cell types of interest is straightforward and achievable. So far, macrophages 38-40 have only been tracked *in vivo* by MR imaging. The hypointense or negative contrast of the MR imaging labeling agent, however, might be difficult to interpret in certain tissues, for example in the lung, or certain tissue conditions such as necrosis. Additionally, the presented labeling approach permits labeling and imaging of cell populations that cannot be targeted specifically *in situ* with radiolabeled antibodies or antibody fragments such as MDSCs. However, it comes with certain drawbacks: direct labeling approaches, such as the antibody-receptor targeting approach described, do not permit monitoring of cell viability or functionality *in vivo*. Furthermore, direct labeling approaches might suffer from label, and hence, signal dilution over time when not terminally differentiated, proliferating cells are labeled. Repetitive imaging is restricted to the biological half-life of the label. Nevertheless, direct labeling approaches are commonly more readily applicable to different cell types of interest than indirect labeling approaches. Indirect labeling approaches rely on genetic modifications, e.g. the introduction of a reporter gene suitable for the imaging modality of choice, of cell types or whole organisms which can be time- and labor-intensive 13, 41, 42. Therefore, our combined PET and MR imaging approach could provide a valuable platform for the imaging of CD11b^+^ cell subsets with high detection sensitivity and spatial resolution.

As discussed above, the direct labeling approach applied to visualize [^64^Cu]PMN- and [^64^Cu]M-MDSC trafficking *in vivo* does not permit interrogation of the cells' state after adoptive transfer into the tumor-bearing hosts. In a previous study, however, we were able to non-invasively image the migration of bone marrow-derived MDSCs to the primary PyMT breast cancer TME. In a subsequent analysis, the fluorescent dye used for fluorescence OI permitted the assessment of the fate of the adoptively transferred MDSCs: the isolated cells were viable or differentiated further into dendritic cells or macrophages. While both viable and further differentiated cells could be detected, fractions of the adoptively transferred MDSCs might have been phagocyted by e.g. macrophages 20. And while it would be highly interesting to examine if the functional capacity of the adoptively transferred MDSCs was changed *in vivo*, it would be challenging to specifically isolate the transferred cells as their phenotype is similar to endogenous MDSCs and functional assessment *ex vivo* with an immunosuppression assay would need high numbers of pure cells.

In the last decade, the frequency of MDSCs in the peripheral blood of tumor-bearing mice or cancer patients was heavily examined and further correlated with disease stage and survival 27, 43, 44. In this study, the experimental separation of the *in vitro* generated MDSCs allowed us to temporally quantify tumor and metastases homing and examine tumor tropism of the subpopulations PMN- and M-MDSCs. In the primary tumor models, the PyMT breast tumors tended to recruit a higher proportion of adoptively transferred [^64^Cu]M-MDSCs while B16F10 primary melanomas were more profoundly infiltrated by [^64^Cu]PMN-MDSCs. Comparably, Toh *et al.* reported PMN-MDSCs as the predominant MDSC subpopulation in the tumors of a transgenic murine melanoma model and further indicated PMN-MDSC-mediated promotion of cancer cell dissemination in this model 45.

Beside the stronger recruitment to the primary tumors, [^64^Cu]PMN-MDSCs showed an increased signal in the *ex vivo* biodistribution analysis of various organs including the blood of the B16F10 melanoma-bearing animals at 48 h post MDSC transfer. This observation prompted the question if survival of [^64^Cu]PMN-MDSCs in tumor-bearing mice was enhanced in comparison to [^64^Cu]M-MDSCs. As G-CSF is an important growth factor promoting steady-state myelopoiesis and, in particular, the production and differentiation of neutrophils 46, the G-CSF plasma levels in PyMT breast tumor- and B16F10 melanoma-bearing mice were analyzed. Interestingly, plasma levels of G-CSF did not differ significantly between PyMT breast cancer- and B16F10 melanoma-bearing mice (Figure S5) indicating that primary B16F10 melanoma tumors recruited higher fractions of [^64^Cu]PMN-MDSCs independently of cell survival.

To further correlate the uptake of adoptively transferred [^64^Cu]PMN-MDSCs and [^64^Cu]M-MDSCs to endogenous MDSCs in the PyMT breast tumors and B16F10 melanoma tumors, flow cytometric profiling of the immune cell infiltrates in these tumors was performed. Interestingly, while B16F10 melanomas showed higher frequencies of both endogenous PMN- and M-MDSCs than PyMT breast tumors, the distribution of the MDSC subpopulations, however, did not correlate exactly with the uptake values of adoptively transferred MDSCs. Adoptively transferred MDSCs were characterized by cell surface marker expression for their phenotypes and an immunosuppression assay for their functionality according to current recommendations before transfer into tumor-bearing mice 6. Profiling for endogenous PMN-MDSCs as CD11b^+^Ly6C^int^Ly6G^+^ cells and endogenous M-MDSCs as CD11b^+^Ly6C^+^Ly6G^-^ cells, however, did not allow for a clear functional discrimination according to current recommendations. As other tumor-resident myeloid cells such as tumor-associated macrophages, are also characterized as CD11b^+^Ly6C^+^Ly6G^-^, we assumed that, in parallel with endogenous M-MDSCs, we further detected a substantial frequency of heterogeneous tumor-associated macrophages 47.

Independent of the MDSC subpopulation, MDSCs fundamentally affect many different stages of tumor invasion and metastasis by, e.g., modulation of the extracellular matrix at the invasive margins and tumor vascularization as well as promotion of metastatic outgrowth 48, 49. Correspondingly, in the examined metastasis models, overall MDSC recruitment was considerably higher than in the corresponding primary tumor models. Additionally, B16F10 melanoma lung metastases recruited a higher fraction of the adoptively transferred [^64^Cu]PMN-MDSCs and [^64^Cu]M-MDSCs than PyMT breast cancer lung metastases.

Moreover, B16F10 primary melanomas and melanoma metastases in the lungs exhibited higher uptake of both [^64^Cu]PMN- and [^64^Cu]M-MDSCs in comparison to primary and metastatic PyMT breast cancer. The difference in the recruitment of radiolabeled MDSC subpopulations to the primary and metastatic TME of these cancer types reflects current literature on MDSC subpopulations in cancer patients: The more aggressively growing B16F10 melanoma model recruited more radiolabeled MDSCs independently of the subpopulation reflecting, to some extend, poor prognosis and overall survival in patients with high frequency of MDSCs in peripheral blood. Interestingly, current literature reports both an increase in the frequency of M-MDSCs in the peripheral blood of patients with malignant melanoma and an overall increase of PMN- and M-MDSCs in melanoma patients 50, 51. Immune checkpoint inhibitor treatment was further correlated with an early decrease in the frequency of peripheral blood PMN-MDSCs in a small melanoma patient cohort 52.

Due to their immunosuppressive nature, MDSCs receive considerable attention as possible negative players in cancer immunotherapeutic approaches 53, 54. While strategies to deplete, to further differentiate MDSCs or to modulate MDSC function in the TME have been published 55-57, newly emerging studies report combinations of MDSC-targeting agents with more and more routinely used immunotherapy approaches 58-60. Clinical *in situ* imaging of MDSC migration in cancer patients with, e.g. immunoPET, however, is challenging due to the lack of an MDSC-specific marker that can be targeted with an immunoPET tracer. Therefore, our molecular imaging approach to visualize MDSC migration and tumor homing *in vivo* could be used to further dissect the effects of combinational therapies and immunotherapeutics on MDSC migration and their capacity to infiltrate tumors or secondary tissues.

## Abbreviations

%ID/cm³: percentage of injected dose per cubic centimeter
%ID/g: percentage of injected dose per gram
[^18^F]FEAU: 2'-deoxy-2'-[^18^F]fluoro-5-ethyl-1-β-D-arabinofuranosyl-uracil
[^18^F]FHBG: 9-(4-[^18^F]fluoro-3-[hydroxymethyl]butyl)guanine
[^64^Cu]M-MDSCs: [^64^Cu]NOTA-αCD11b-mAb-labeled M-MDSCs
[^64^Cu]PMN-MDSCs: [^64^Cu]NOTA-αCD11b-mAb-labeled PMN-MDSCs
[^64^Cu]PTSM: [^64^Cu]pyruvaldehyde bis(N4-methylthiosemicarbazone)
7-AAD: 7-aminoactinomycin D
ATCC: American type culture collection
CD11b-M-MDSCs: αCD11b-mAb labeled M-MDSCs
CD11b-PMN-MDSCs: αCD11b-mAb labeled PMN-MDSCs
CFSE: carboxyfluorescein succinimidyl ester
eMDSCs: early-stage MDSCs
FCS: fetal calf serum
G-CSF: granulocyte colony stimulation factor
GM-CSF: granulocyte-macrophage colony stimulation factor
HSV1-tk: herpes simplex virus-1 thymidine kinase
i.c.: intracardiac
IFN-γ: interferon γ
IL-6: interleukin 6
i.v.: intravenous
Luc: Luciferase
mAb: monoclonal antibody
M-CSF: macrophage colony stimulation factor
MDSCs: myeloid-derived suppressor cells
M-MDSC: monocytic myeloid-derived suppressor cells
MRI: magnetic resonance imaging
NOTA: 1,4,7-triazacyclononane-triacetic acid
NOTA-NHS: NOTA-N-hydroxysuccinimide ester
OI: optical imaging
PBS: phosphate buffered saline
PET: positron emission tomography
PMN-MDSCs: polymorphonuclear myeloid-derived suppressor cells
PyMT: polyoma middle T antigen
SPIOs: superparamagnetic iron oxide particles
TGF- β: transforming growth factor β
TME: the tumor microenvironment
TNF: tumor necrosis factor
VOI: volume of interest
