# Supplementary material for: Visualization and quantification of in vivo homing kinetics of myeloid-derived suppressor cells in primary and metastatic cancer
Source: Theranostics. 2019 Aug 12;9(20):5869–85. doi: 10.7150/thno.33275 (PMC6735369; doi:10.7150/thno.33275)
Supplement: Supplementary file 1 — Supplementary figures and tables. [file thnov09p5869s1.pdf]

Supplementary Figure 1

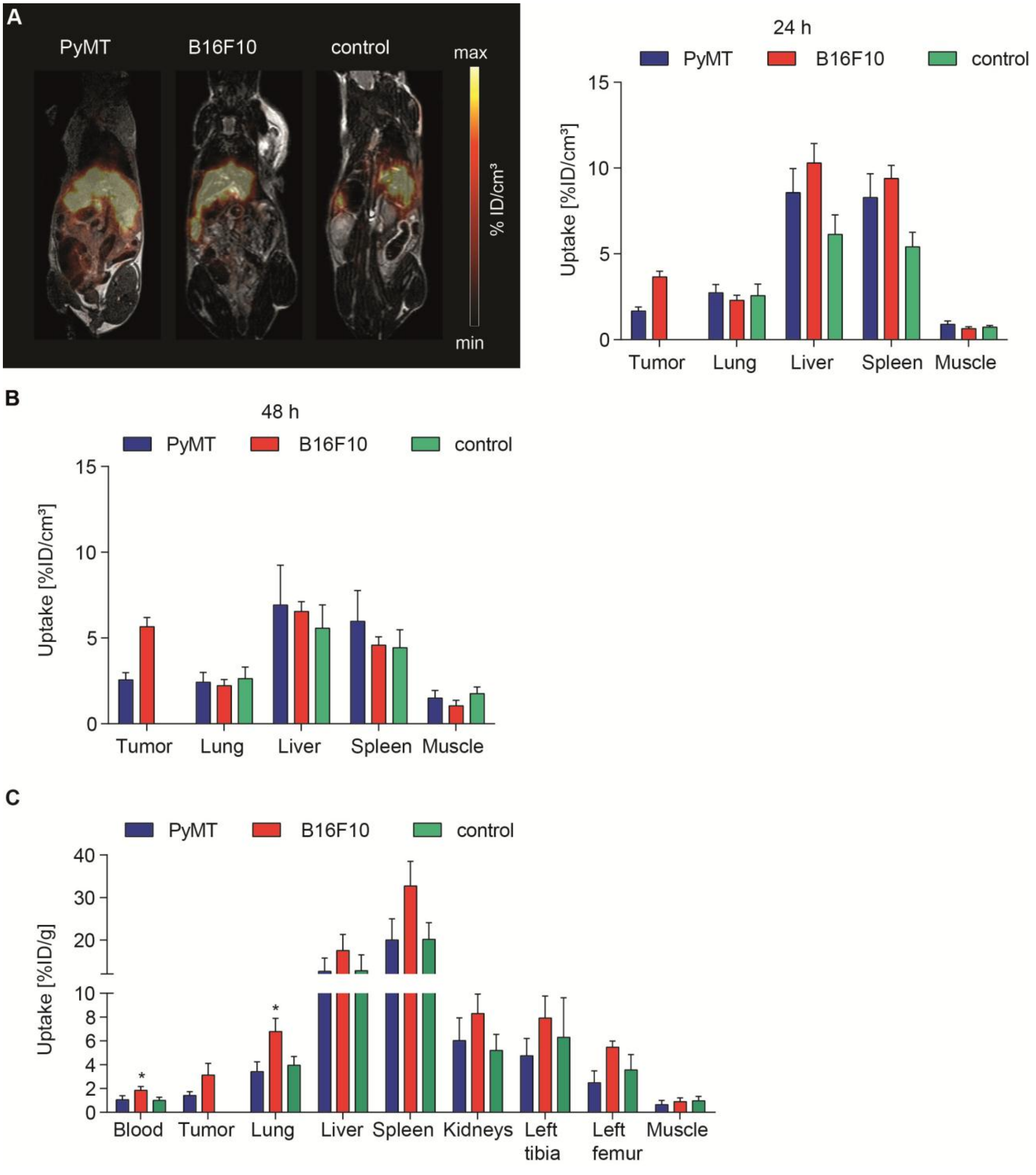

Supplementary figure 1: Exemplary whole-body PET/MR fusion images, *in vivo* and *ex vivo* biodistribution of [^{64}Cu]PMN-MDSCs in the PyMT breast and B16F10 melanoma primary tumor models and naïve littermate controls. Exemplary PET/MRI fusion images (**A**, left panel) and image-derived quantification of the *in vivo* biodistribution (**A** , right panel) of [^{64}Cu]PMN-MDSCs revealed homing to the primary tumor as early as 24 h post adoptive transfer with enhanced homing at 48 h post adoptive transfer (**B**). Other sites of PMN-MDSC migration include the lung, liver and spleen of tumor-bearing animals while muscle is given for comparison. The *ex vivo* biodistribution by γ-counting (**C**) confirmed [^{64}Cu]PMN-MDSCs homing to the primary PyMT breast tumor and B16F10 melanoma as well as higher uptake values in the primary B16F10 melanoma at 48 h post adoptive cell transfer. Furthermore, significantly higher proportions of [^{64}Cu]PMN-MDSCs were found in the blood and lungs of B16F10 melanoma-bearing animals in comparison to naïve littermate controls. Values are given as mean ± SEM in %ID/cm^3 for (**A**) and (**B**) and as mean ± SEM in %ID/g for (**C**), n=5 for PyMT, n=6 for B16F10, n=5 controls, statistics: Dunnett's Multiple Comparison Test, *$p<0.05$.

Supplementary Figure 2

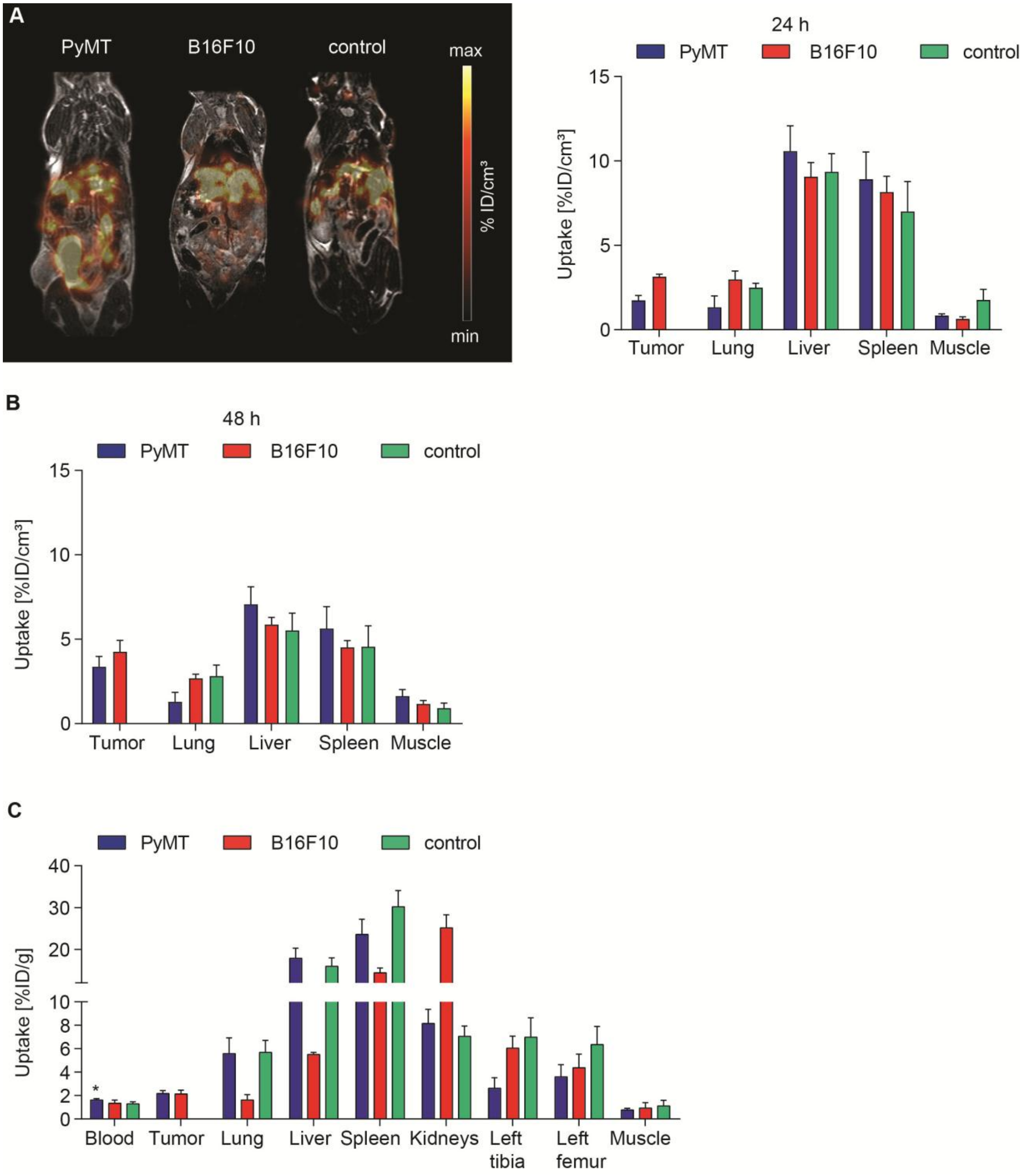

Supplementary figure 2: Exemplary whole-body PET/MR fusion images, *in vivo* and *ex vivo* biodistribution of [^{64}Cu]M-MDSCs in the PyMT breast and B16F10 melanoma primary tumor models and naïve littermate controls. Exemplary PET/MR fusion images (**A**, left panel) and image-derived quantification of the *in vivo* biodistribution of [^{64}Cu]M-MDSCs at 24 h (**A**, right panel) and 48 h (**B**) post adoptive transfer revealed homing to the primary PyMT breast tumor and B16F10 melanoma. Beside the primary tumors, other sites of [^{64}Cu]M-MDSC migration include the lung, liver and spleen while the muscle is given for comparison. The *ex vivo* biodistribution by γ-counting (**C**) confirmed [^{64}Cu]M-MDSCs homing to the primary PyMT breast tumor and B16F10 melanoma. In addition, in PyMT breast cancer tumor-bearing animals, a significantly higher proportion of [^{64}Cu]M-MDSCs remained in the blood in comparison to naïve littermate controls. Values are given as mean ± SEM in %ID/cm³ for (**A**) and (**B**) and as mean ± SEM in %ID/g for (C), n=6 for PyMT, n=5 for B16F10, n=5 controls, statistics: Dunnett's Multiple Comparison Test, *$p<0.05$.

Supplementary Figure 3

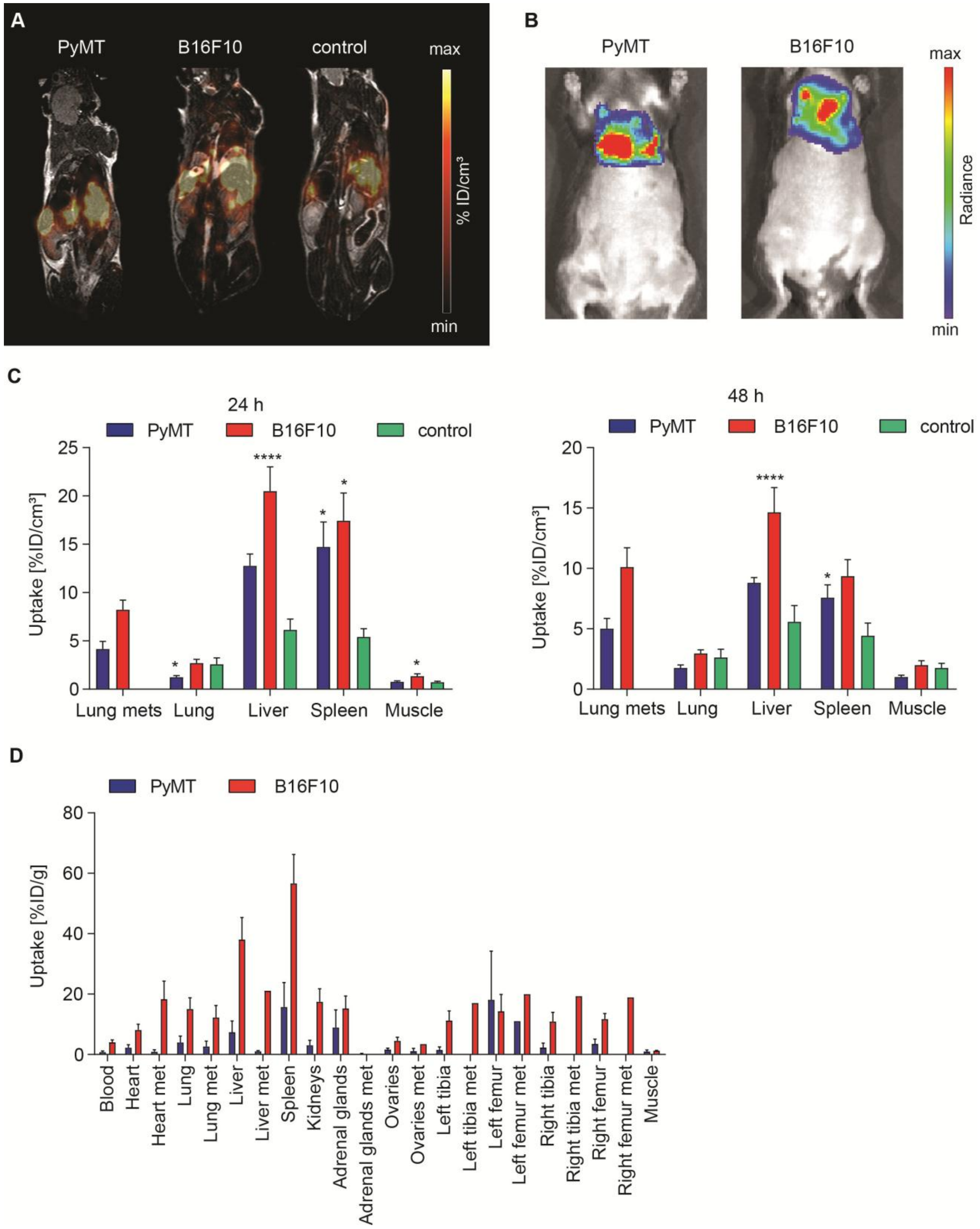

Supplementary figure 3: Exemplary whole-body PET/MR fusion and bioluminescence OI images, *in vivo* and *ex vivo* biodistribution of [^{64}Cu]PMN-MDSCs in the PyMT breast cancer and B16F10 melanoma metastasis models and naïve littermate controls.

Representative PET/MR fusion images (**A**) of [^{64}Cu]PMN-MDSC whole-body distribution *in vivo* at 24 h post adoptive cell transfer and bioluminescence OI images (**B**) visualizing the PyMT breast cancer and B16F10 melanoma metastatic lesions at 48 h post adoptive cell transfer. The image-derived quantification of the *in vivo* distribution of [^{64}Cu]PMN-MDSCs revealed homing to lung metastatic lesions (denoted lung mets) at 24 h (**C**, left panel) and 48 h (**C**, right panel) post cell injection and higher uptake values than in control healthy lung tissue. However, small lung metastases could not be identified in the *in vivo* PET/MR fusion images and were consequently not taken into account for quantification. Due to technical difficulties in the dissection of small lung metastases, the higher uptake of [^{64}Cu]PMN-MDSCs in lung metastatic lesions could not be verified in the *ex vivo* quantification by γ-counting (**D**). Values are given as mean ± SEM in %ID/cm³ for (**C**), as mean ± SEM in %ID/g for (**D**), n=7 for PyMT, n=6 for B16F10, n=5 controls, statistics: Dunnett's Multiple Comparison Test, *p<0.05, ****p<0.001.

Supplementary Figure 4

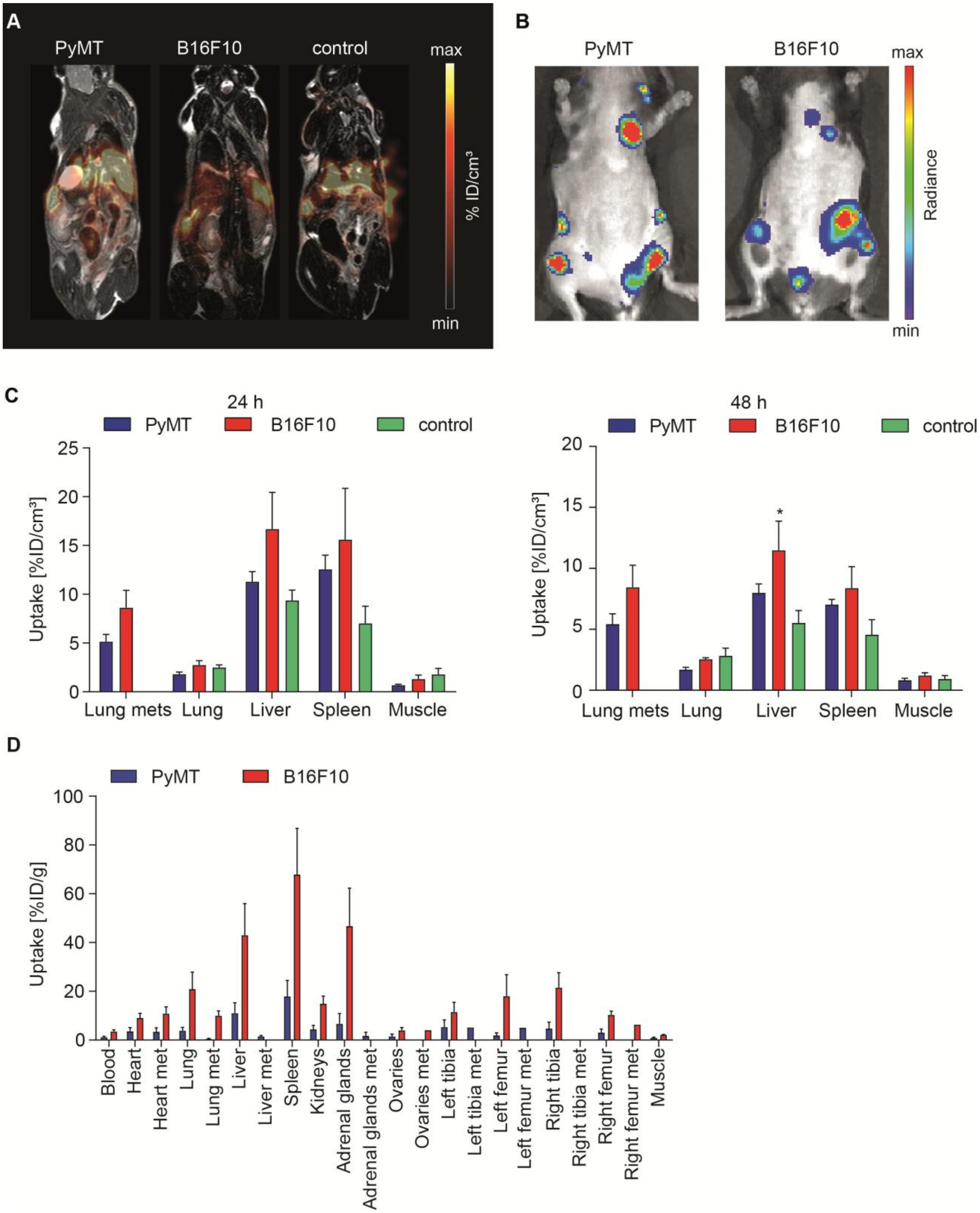

Supplementary figure 4: Exemplary whole-body PET/MR fusion images and bioluminescence OI images, *in vivo* and *ex vivo* biodistribution of [^{64}Cu]M-MDSCs in the PyMT breast cancer and B16F10 melanoma metastasis models and naïve littermate controls.

Exemplary PET/MR fusion images (**A**) of [^{64}Cu]M-MDSC whole-body distribution *in vivo* at 24 h and bioluminescence OI images (**B**) visualizing the PyMT breast cancer and B16F10 melanoma metastatic lesions at 48 h post adoptive cell transfer. Image-derived quantification of the *in vivo* biodistribution of [^{64}Cu]M-MDSCs at 24 h (**C**, left panel) and 48 h (**C**, right panel) after adoptive cell transfer revealed higher uptake values in lung metastases (denoted lung mets) than in control healthy lung tissue. The quantification of small lung metastases, however, was not feasible from *in vivo* PET data and was therefore not taken into account for quantification. Furthermore, due to technical difficulties in the dissection of small lung metastases, the higher uptake of [^{64}Cu]M-MDSCs in lung metastatic lesions could not be verified in the *ex vivo* quantification by γ-counting (**D**) since not all metastases could be dissected to be measure separately from healthy lung tissue. Values are given as mean ± SEM in %ID/cm^3 for (**C**), as mean ± SEM in %ID/g for (**D**), n=6 for PyMT, n=5 for B16F10, n=5 controls, statistics: Dunnett's Multiple Comparison Test, *$p<0.05$.

Supplementary figure 5

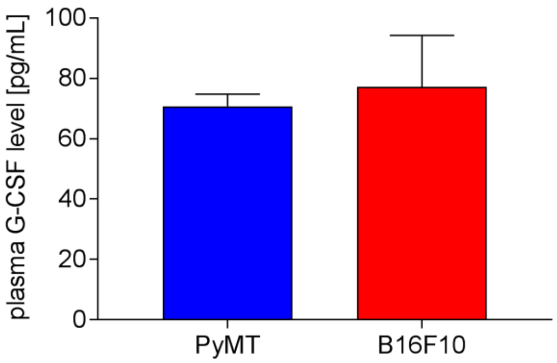

Supplementary figure 5: Plasma G-CSF levels in primary PyMT breast cancer- and primary B16F10 melanoma-bearing mice.
G-CSF levels in the plasma of PyMT breast cancer- and B16F10 melanoma-bearing mice did not differ significantly indicating no direct effect of G-CSF on the difference of [^{64}Cu]PMN-MDSC and [^{64}Cu]M-MDSC levels in the peripheral blood observed in the cell tracking experiments (n=4, values are given as mean ± SEM in pg/mL, statistics: Student's t-test).
